# Sustainable High‐Performance Aqueous Batteries Enabled by Optimizing Electrolyte Composition

**DOI:** 10.1002/advs.202417587

**Published:** 2025-05-05

**Authors:** Raphael L. Streng, Samuel Reiser, Anatoliy Senyshyn, Sabrina Wager, Johannes Sterzinger, Peter Schneider, David Gryc, Mian Zahid Hussain, Aliaksandr S. Bandarenka

**Affiliations:** ^1^ Physics of Energy Conversion and Storage Department of Physics Technische Universität München (TUM) James‐Franck‐Str. 1 85748 Garching Germany; ^2^ Heinz Maier‐Leibnitz Zentrum (MLZ) Technische Universität München Lichtenbergstr. 1 85748 Garching Germany; ^3^ Chair of Inorganic and Metal‐Organic Chemistry Department of Chemistry School of Natural Sciences TUM, Lichtenbergstraße 4 85748 Garching Bavaria Germany; ^4^ Catalysis Research Center TUM Ernst‐Otto‐Fischer‐Straße 1 85748 Garching Germany

**Keywords:** aqueous batteries, electrolytes, fast charging, magnesium, potassium

## Abstract

Lithium‐free aqueous batteries (LFABs) offer a sustainable alternative to lithium‐ion batteries for large‐scale energy storage, addressing issues like material scarcity and flammability. However, their economic viability is limited by low energy density and cycle life due to the narrow electrochemical stability window of water and active material dissolution. High‐concentration water‐in‐salt electrolytes typically used to tackle these issues are expensive and potentially hazardous. This work presents a novel, cost‐efficient electrolyte design using safe salts at lower concentrations. The influence of different cation species on the copper hexacyanoferrate cathode and polyimide anode is systematically explored, optimizing the electrolyte for improved cell voltage and cycling stability. The resulting battery, with a 1.8 mol kg^⁻1^ MgCl_2_ + 1.8 mol kg⁻^1^ KCl aqueous electrolyte, achieves a competitive energy density of 48 Wh kg⁻¹ and 95% efficiency. It also shows 70% capacity retention even at extremely high (dis‐)charge rates of 50 C and a maximum specific power of over 10000 W kg⁻¹, indicating its strong potential for supercapacitor applications. Utilizing exclusively inexpensive and safe salts, this work significantly advances the practical application of low‐cost LFABs for large‐scale energy storage.

## Graphical abstract

Lithium‐free aqueous batteries provide a safe, low‐cost alternative to lithium‐ion batteries for energy storage. This study introduces an optimized electrolyte using inexpensive salts, enhancing both energy density and cycling stability. The resulting battery delivers high power and efficiency, demonstrating strong potential for supercapacitor applications and large‐scale energy storage.

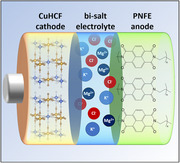

## 1 Introduction

The ongoing transition from fossil fuels toward renewable energy sources and the electrification of the transport sector necessitate novel energy storage solutions.^[^ ^1^, ^2^, ^3^, ^4^ ^]^ Especially in mobile applications and large‐scale stationary storage, batteries have taken a central role in the last decades.^[^ ^5^ ^]^ Lithium‐ion batteries (LIB) are predominant in battery electric vehicles (BEV) and mobile devices within the wide range of battery systems.^[^ ^6^ ^]^ While their unmatched energy density justifies their leading position in the market, their inherent safety concerns and the scarcity of lithium motivate an increasing interest in beyond‐lithium‐ion batteries.^[^ ^7^, ^8^ ^]^ Cell chemistries based on, e.g., sodium, potassium, magnesium, or calcium, can be a more sustainable and cost‐efficient alternative to conventional LIBs. These metals are up to three orders of magnitude more abundant in the earth's crust than lithium, making them easily accessible in most parts of the world.^[^ ^9^ ^]^ In addition, safety concerns related to flammability and toxicity can be overcome by using aqueous instead of organic electrolytes. Therefore, lithium‐free aqueous batteries (LFAB) have recently received growing attention.^[^ ^10^, ^11^ ^]^ While the narrow electrochemical stability of water limits their cell voltage and, thus, energy density, they offer advantageous properties for large‐scale energy storage. In addition, the high ionic conductivity of aqueous electrolytes enables ultrahigh power densities, making them a viable choice for applications requiring fast‐charging capabilities.^[^ ^12^, ^13^ ^]^ These include fast‐response stationary storage devices for grid stabilization and applications in the transport sector. Whereas personal vehicles optimized for long‐range rely on LIBs, public transport can significantly benefit from high‐power‐density systems.^[^ ^14^, ^15^ ^]^ Multiple companies have developed buses using supercapacitors (SCs) that can be charged quickly during normal operation.^[^ ^16^, ^17^ ^]^ In this sector, aqueous battery‐SC‐hybrid systems can be a promising alternative, offering higher energy densities than SCs and better fast‐charging capabilities than LIBs.

However, LFABs have not been successfully implemented into the market so far, as their economic viability remains doubtful due to considerably lower energy densities compared to state‐of‐the‐art LIBs.^[^ ^18^, ^19^ ^]^ Thus, the narrow electrochemical stability window of water and the limited cell voltage can be regarded as the main drawback of aqueous batteries.^[^ ^20^ ^]^ In addition, rapid capacity degradation due to active material dissolution has been identified as one of the main challenges to the practical application of LFABs.^[^ ^21^, ^22^ ^]^ A commonly used strategy to address these issues is the use of highly concentrated water‐in‐salt electrolytes (WiSE). By minimizing the amount of “free water” through molecular crowding, water‐splitting side reactions and electrode material hydrolysis can be mitigated effectively.^[^ ^23^ ^]^ Since the original WiSE, 21 mol kg^−1^ LiTFSI, was proposed by Suo et al. in 2015, multiple alternative water‐in‐salt systems have been identified.^[^ ^24^ ^]^ In particular, KN(SO_2_F)_2_ (KFSI) and KSO_3_CF_3_ (KOTf) are frequently used due to their exceptional solubilities. In 2019, Jiang et al. reported a high energy density aqueous battery based on 22 m KOTf,^[^ ^25^ ^]^ and in 2020, a 62 mol kg^−1^ aqueous KFSI/KOTf electrolyte was developed by Ko et al.^[^ ^26^ ^]^ Although effective, this approach depends on costly and toxic fluorinated salts, which raise the per‐Wh cost and increase safety risks for the battery. Hence, WiSEs based on more available salts have been the subject of extensive research in recent years. The most promising candidates include potassium acetate (KAc)^[^ ^27^ ^]^ and NaClO_4_,^[^ ^28^ ^]^ which come at significantly lower prices than the systems mentioned above. However, the high basic strength of acetates limits their compatibility with certain commonly used electrode materials, such as Prussian blue analogs (PBAs).^[^ ^29^ ^]^ At the same time, the strongly oxidative nature of perchlorates is linked to potentially significant safety risks, especially at concentrations close to the solubility limit. In addition, various organic and inorganic nonionic additives, such as sugar or metal oxides, have been demonstrated to show great potential as molecular crowding agents for aqueous electrolytes, suppressing side reactions and degradation.^[^ ^30^, ^31^ ^]^

In this work, we present a novel approach to tuning important electrode properties by systematically investigating the effect of the cation on the redox potential and the cycling stability of a copper hexacyanoferrate (CuHCF) cathode and a polyimide anode. Namely, sodium, potassium, magnesium, and calcium ions were used as they represent the most abundant alkali and alkaline earth metals in the earth's crust and are, thus, the most promising candidates for designing low‐cost and sustainable batteries.^[^ ^9^ ^]^ Based on these findings, we propose a relatively low‐concentration mixed‐salt electrolyte enabling an optimized cell voltage and battery cycle life. Using only highly abundant KCl and MgCl_2_ in the electrolyte, the model cell achieves a competitive energy density and a remarkable fast‐charging capability and power density, representing a significant step toward a practical and cost‐efficient design for aqueous batteries.

## 2 Results and Discussion

**Figure 1:**
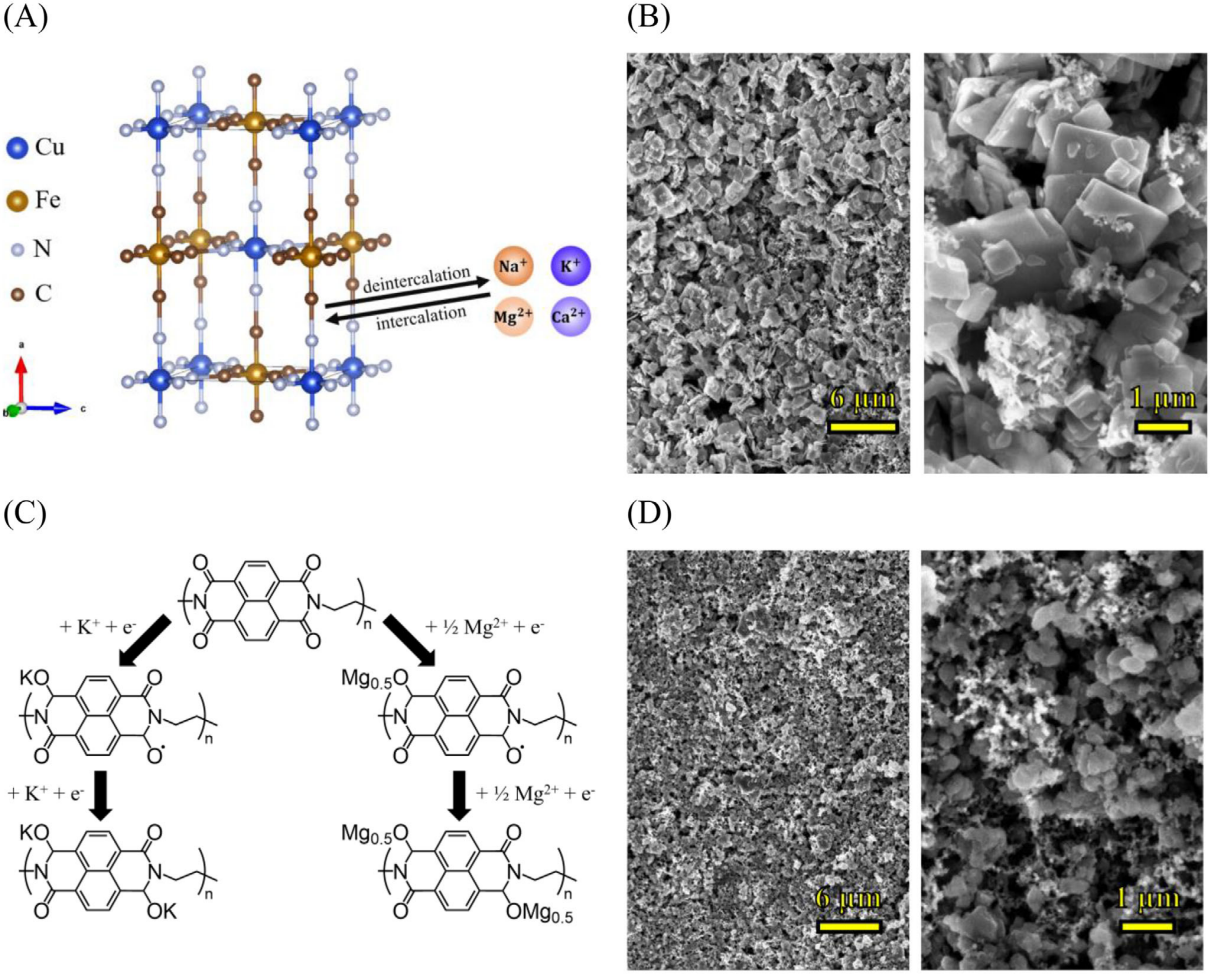
Charge storage mechanisms and structures of the anode and cathode. A) Intercalation mechanism and crystal structure of CuHCF. B) SEM images of the CuHCF cathode. C) Enolization mechanism and chemical structure of PNFE. D) SEM images of the PNFE anode.

Prussian blue analogs (PBAs) are among the most promising active cathode materials for aqueous batteries. Their open‐channel structure and large interstitial sites enable exceptional fast‐charging capabilities, allowing them to host even large cations such as Na^+^ and K^+^.^[^ ^32^, ^33^ ^]^ Furthermore, the facile synthesis, compositional and electrochemical tunability, and the abundance of commonly used precursor materials make them a particularly interesting group of materials for grid‐scale energy storage. One of the most investigated PBAs is copper hexacyanoferrate (CuHCF), which offers a high electrode potential close to the oxygen evolution threshold and has been shown to host a multitude of monovalent and multivalent cations.^[^ ^34^, ^35^, ^36^ ^]^ As shown in our previous work, the charge‐storage mechanism of CuHCF features at least three distinct stages. Based on impedance spectroscopy measurements, these stages are detectable for the intercalation of monovalent and divalent cations. For a more detailed analysis of the intercalation mechanism, the reader is referred to our previous studies.^[^ ^37^ ^]^ Generally, CuHCF is synthesized via a simple coprecipitation reaction from two aqueous solutions containing Cu ions and Fe(CN)_6_ ions, respectively. However, this synthesis route leads to irregular particles with low crystallinity and high amounts of interstitial water, impairing the specific capacity.^[^ ^38^ ^]^ Hence, chelating agents like sodium citrate or sodium pyrophosphate have been established as a reliable method to minimize vacancies and water content.^[^ ^39^, ^40^ ^]^ In this work, CuHCF was synthesized according to the method reported by Xu et al., which used sodium pyrophosphate and polyvinyl pyrrolidone as chelating additives.^[^ ^41^ ^]^ In their work, Xu et al. found that the so‐prepared highly crystalline CuHCF features a lower water content with increased Na‐storage capabilities compared to the conventional simple coprecipitation method. The fitted X‐ray diffraction (XRD) pattern of the resulting particles is shown in Figure S1 (Supporting Information). Interestingly, this synthesis modification leads to a change from the typical cubic crystal structure of PBAs to a monoclinic structure with lattice parameters *a* = 11.22 Å, *b* = 7.30 Å, and *c* = 6.95 Å, which is depicted in **Figure** **1**A,B shows a scanning electron microscopy (SEM) picture of the cathode used in this work, consisting of sheet‐shaped CuHCF crystals in the 200–800 nm size range. The pore structure of the cathode was analyzed using Brunauer–Emmett–Teller (BET) analysis (Figure S3A, Supporting Information). The N_2_ adsorption exhibits a Type II isotherm, which indicates the nonporous (or macroporous) nature of the sample. A minimal uptake at *p*/*p*₀ < 0.2 suggests weak interactions between N_2_ molecules and the surface, confirming the absence of micro/mesopores. In the mid‐pressure range (*p*/*p*₀ = 0.2–0.8), multilayer adsorption dominates, followed by a steep rise in adsorption at *p*/*p*₀ > 0.9, which is attributed to N_2_ condensation in macropores or on the external surface of the material. The BET specific surface area (SSA) of the CuHCF cathode was calculated to be 1.9 m^2^ g^−1^, which is extremely low, further confirming the macroporous nature or low accessible surface area. The graphite substrate, which makes up the larger part of the overall cathode mass, showed a negligible SSA of 0.5 m^2^ g^−1^ under the same measurement conditions.

Besides PBAs, redox‐active organic compounds have gained recent interest, particularly as anode materials for LFABs, owing to their sustainability and electrochemical tunability.^[^ ^42^, ^43^, ^44^ ^]^ Especially, materials based on perylene diimide and naphthalene diimide (NDI) have been successfully implemented as anodes for various monovalent and multivalent ion aqueous batteries.^[^ ^25^, ^45^ ^]^ Our previous work investigated the charge storage mechanism and exceptional electrochemical performance of poly(naphthalene four formyl ethylenediamine) (PNFE), an NDI‐derived redox‐active polymer.^[^ ^46^ ^]^ Through an enolization process shown in Figure 1A, the polyimide can store various monovalent and divalent ions such as Na^+^, K^+^, and Mg^2+^. This material was selected as the anode for this work because it offers superior cation storage capacity and fast‐charging capabilities. PNFE, synthesized for our previous study, is formed in a one‐step dehydration condensation reaction between 1,4,5,8‐naphthalene tetracarboxylic dianhydride and ethylene diamine. Figure S2A (Supporting Information) shows the Fourier‐transform infrared (FTIR) spectrum of the as‐prepared polymer, confirming the successful synthesis as the observed peaks are in good accordance with the expected chemical structure. Stretching vibrations of the electrochemically active carbonyl groups correspond to the bands observed at 1700 and 1661 cm^−1^. The other peaks can be assigned to vibrations of C═C bonds in the cyclic alkenes (1580 cm^−1^), various vibrations of aromatic and nonaromatic C─N bonds (1347 to 1094 cm^−1^), and vibrations of C─H bonds (1452, 1374, 1031, and 768 cm^−1^), respectively. An X‐ray photoelectron spectroscopy (XPS) survey scan and the fitted C1s spectrum of the electrode slurry coated onto a pyrolytic graphite sheet replotted from our previous work are shown in Figure S2B,C (Supporting Information).^[^ ^46^ ^]^ Characteristic peaks for F, O, N, and C can be observed, confirming the presence of both the active material and the Nafion binder at the electrode surface. In the C1s spectrum, peaks related to C═C, C─C, C─N, N─C═O, C═O, CF_2_, and CF_3_ bonds, as well as a π–π* satellite, are found. Figure 1D (Supporting Information) shows an SEM picture of the anode, revealing a porous structure with particle sizes around 100 nm and pores mostly in the micrometer range. This was confirmed by BET analysis (Figure S3B, Supporting Information). Like the cathode, the N_2_ adsorption of the PNFE anode exhibits a Type II isotherm, indicating a microporous nature. It shows a similar behavior as the cathode with a specific area of 1.5 m^2^ g^−1^.

**Figure 2:**
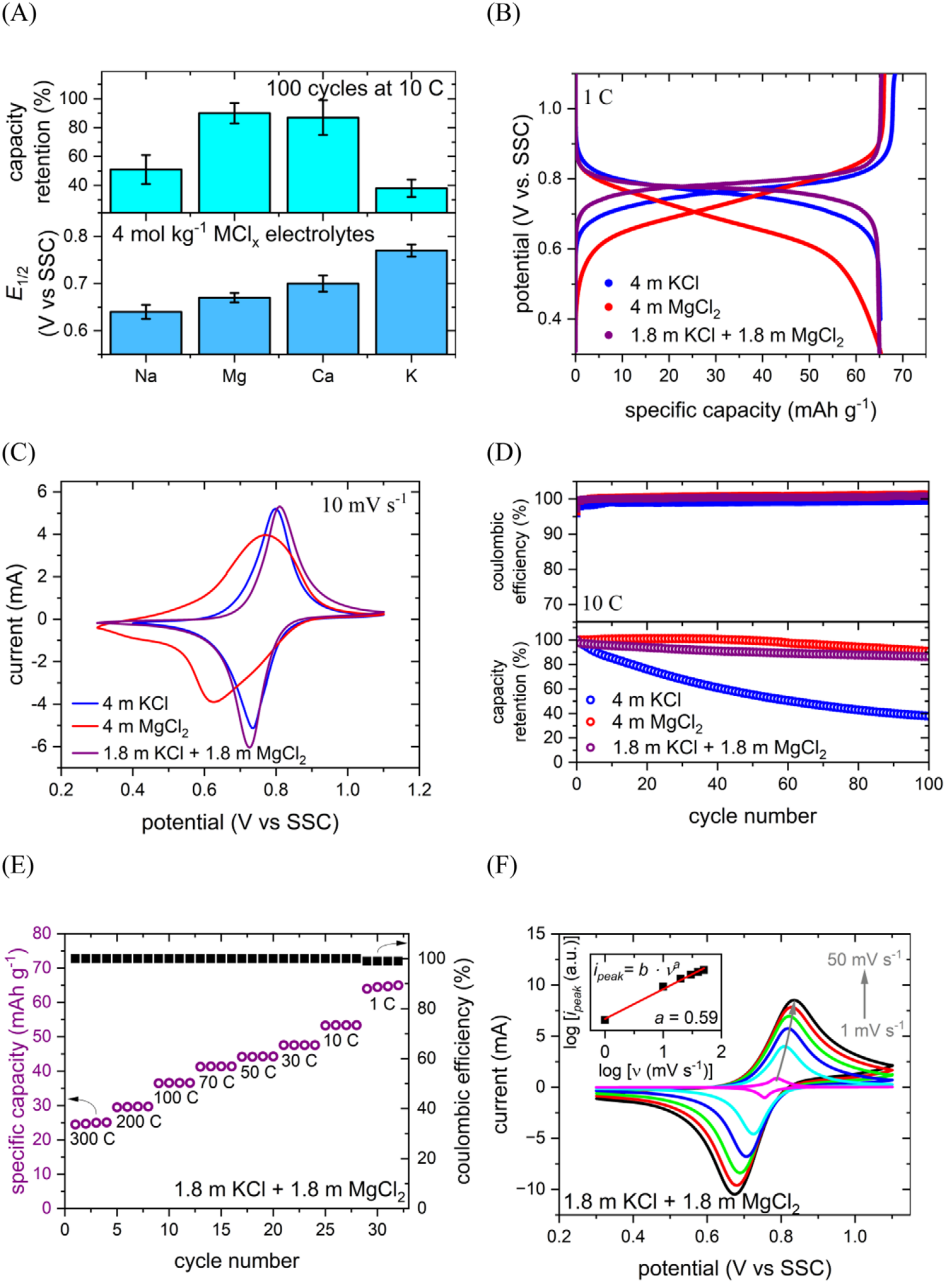
Electrochemical performance of CuHCF. A) Summary of the electrode potential and cycling stability in different 4 mol kg^−1^ MCl_x_ (M = Na, K, Mg, Ca) aqueous electrolytes. Data presented as mean ± SD, *n =* 5. B) Galvanostatic (dis‐)charge curves, C) CVs, and D) cycling stability in KCl, MgCl_2_, and 1.8 mol kg^⁻1^ KCl + 1.8 mol kg^⁻1^ MgCl_2_. E) Specific discharge capacity in 1.8 mol kg⁻^1^ KCl + 1.8 m MgCl_2_. F) CVs at different scan rates in 1.8 mol kg^⁻1^ KCl + 1.8 mol kg^⁻1^ MgCl_2_.

As mentioned above, both electrode materials can host a large variety of alkali metal and alkaline earth metal cations, of which Na^+^, K^+^, Mg^2+^, and Ca^2+^ can be regarded as the most interesting for designing sustainable battery systems. Hence, the influence of the cation species on important electrode properties was investigated by cyclic voltammetry and galvanostatic cycling in 4 mol kg^−1^ MCl_x_ (M = Na, K, Mg, Ca) aqueous electrolytes. **Figure** **2** shows the influence of the electrolyte composition on the electrochemical performance of the CuHCF cathode. A high electrode potential and minimal active material degradation are favorable for cathode materials. Thus, the half charge potential *E*_1/2_ of the cathode in the respective electrolyte was evaluated as the mid‐point between the cathodic and the anodic peak in the cyclic voltammograms (CVs). The cycling stability was assessed by charging and discharging the electrode for 100 cycles at 10 C.

In Figure 2A, the results of both investigations are summarized. As shown in the lower half of the graph, the cathode potential is strongly dependent on the cation choice as it increases by more than 100 mV following the trend Na^+^ < Mg^2+^ < Ca^2+^ < K^+^. Previous studies have shown a strong correlation between the hydration energy of alkali metal cations and the intercalation potential of PBAs.^[^ ^47^, ^48^ ^]^ The smaller hydration shell of K^+^ facilitates the intercalation, thus raising the reduction potential compared to Na^+^. According to this trend, the significantly more negative hydration energy and larger hydration shells of divalent Mg and Ca ions should lead to lower electrode potentials. However, the intercalation of Mg^2+^ and Ca^2+^ occurs, in fact, at a more positive potential than for Na^+^, which is in accordance with previous literature.^[^ ^49^, ^50^ ^]^ Mizuno et al., who reported similar intercalation potentials for Na^+^ and Mg^2+^ at NiHCF, attributed this effect to the partial dehydration of Mg^2+^ upon intercalation. They found that Mg^2+^ intercalation at the PBA surface is blocked in an organic propylene carbonate (PC) electrolyte. As the PC molecule is too large to co‐intercalate, the divalent ions must be completely desolvated during the charge transfer. However, this leads to strong coulombic repulsion of the bare Mg‐ions at the PBA surface. In aqueous electrolytes, in contrast, Mg^2+^ is only partially dehydrated at the interface enabling Mg‐intercalation in these systems. This significantly lowers the energy demand for intercalation compared to complete dehydration, thus promoting the reduction of the PBA.^[^ ^50^ ^]^ Based on these results, aqueous electrolytes using potassium salts lead to the most positive electrode potentials for CuHCF, enabling the highest energy densities.

However, the stability measurements shown in the upper half of Figure 2A reveal that the cathode active material suffers from severe degradation in both 4 mol kg^⁻1^ NaCl and 4 mol kg^⁻1^ KCl, with only ≈40% of the capacity remaining after 100 cycles at 10 C. This capacity loss may be linked to multiple degradation mechanisms for CuHCF and PBAs that have been suggested and investigated in the past. In summary, three main processes have been identified as the main drivers of PBA degradation upon cycling:

1. Fe(CN)_6_ vacancies introduced during synthesis causing lattice defects have been shown to promote the collapse of the crystal structure during (de)intercalation of cations.^[^ ^51^, ^52^ ^]^ However, this appears unlikely as the governing degradation mechanism for the CuHCF nanosheets as they are characterized by high crystallinity and few defects.^[^ ^41^ ^]^
2. Besides Fe(CN)_6_ vacancies, interstitial or zeolitic water inclusions can form during the synthesis in aqueous media, which also contribute to the degradation of the PBA structure.^[^ ^53^ ^]^ Interstitial water occupying the alkali metal sites can lead to capacity decay through hydrolysis of the PBA framework or block the ion channels, impeding solid‐state diffusion.^[^ ^54^ ^]^ Although the use of sodium pyrophosphate as a chelating agent should help minimize the water content in the structure, a certain amount of interstitial H_2_O is always present in PBAs.
3. Finally, the detrimental effect of OH^−^ species at the electrode surface has been identified as one of the main drivers for electrode degradation for PBAs.^[^ ^29^ ^]^ Naturally, the concentration of OH^−^ in neutral electrolytes such as NaCl and KCl is relatively low. However, a certain amount of H_3_O^+^ and OH^−^ is generally present in any aqueous solution. At operating potentials close to or even beyond the oxygen evolution onset, specific adsorption of OH^−^ on the electrode surface can induce locally increased pH values, promoting the well‐understood alkaline degradation pathway for PBAs. As CuHCF is cycled to potentials well above 1.23 V versus SHE, this effect can contribute substantially to electrode degradation.

The latter two mechanisms are likely responsible for the relatively rapid capacity loss of CuHCF in aqueous KCl and NaCl electrolytes. However, as shown in Figure 2A, only a minimal capacity decay can be observed in electrolytes based on Mg^2+^ or Ca^2+^, indicating that the divalent cations suppress these processes. While the interstitial water content in CuHCF is initially the same for all investigated systems, the higher charge density ions may capture residual water, impeding its negative effects. A similar observation has previously been reported by Xie et al., who achieved significantly enhanced cycle life for a PBA cathode by using AlCl_3_ as an electrolyte additive. They demonstrated that the Al^3+^ ions could bind with interstitial water molecules, removing them from the alkali metal sites and suppressing detrimental side reactions.^[^ ^55^ ^]^ A similar effect can also be assumed for Ca^2+^ and particularly Mg^2+^, which are characterized by high charge densities and, thus, strong interactions with water. Furthermore, smaller divalent ions feature a high reactivity with OH ions, decreasing their activity in aqueous solutions by undergoing a hydrolysis reaction with water:^[^ ^56^, ^57^ ^]^

(1) $$M{{\left(H_{2}O\right)}_{n}}^{2+}\to \mathrm{MOH}{{\left(H_{2}O\right)}_{n-1}}^{+}+H^{+}\left(\mathrm{with}M=\mathrm{Mg},\mathrm{Ca}\right)$$

Consequently, the presence of Mg^2+^ or Ca^2+^ can additionally suppress the alkaline degradation pathway, as OH^−^ species adsorbing at the electrode surface or forming even within the crystal structure from interstitial water are captured. It should also be noted that molecular crowding of the electrolyte has been demonstrated as an effective strategy to improve the cycling stability of PBAs.^[^ ^21^, ^26^ ^]^ Due to their much more negative hydration enthalpy, Mg^2+^ and Ca^2+^ form larger and more strongly bound hydration shells in water, than Na^+^ and K^+^. For example, Mg^2^⁺ typically coordinates with six water molecules in its primary hydration shell, forming an octahedral structure. The Mg─O bond distance in this configuration is ≈2.1 Å. The high charge density of Mg^2^⁺ leads to a well‐ordered and stable hydration shell. This strong interaction also influences the second hydration shell, contributing to the overall stability of the hydrated ion complex.^[^ ^58^, ^59^ ^]^ In contrast, K^+^ ions form a relatively unstable first coordination sphere with a coordination number between 6 and 8 and a K─O distance of ≈2.8 Å. K^+^ ions typically do not form a second coordination sphere.^[^ ^60^ ^]^ Therefore, the strong hydration of multivalent ions decreasing the free water content in the electrolyte likely also contributes to the stabilization of the cathode.

To further analyze the degradation process, the structural changes to the CuHCF cathode after cycling were investigated using SEM and XRD. Figure S5A (Supporting Information) shows the XRD patterns of the fully reduced CuHCF cathode recorded before and after cycling in KCl and MgCl₂.

The initial XRD pattern of the cathode before cycling corresponds to the expected superimposing of CuHCF and the graphite‐based substrate. However, after cycling in either electrolyte, most characteristic reflections of the monoclinic CuHCF structure disappear, leaving the substrate‐dominated pattern. Only the monoclinic (011) (pseudo‐cubic (200)) reflection at 17.7° 2θ remains visible, corresponding to a pseudo‐cubic cell volume of ≈1000 Å^3^ for all samples (Figure S5B, Supporting Information). The reflection FWHM and its position remain nearly unaffected by electrochemical treatment, revealing the coherent scattering domain to be preserved along with the stable chemical composition.

However, the absence of other characteristic peaks suggests extreme abnormal broadening at higher diffraction angles, likely due to high microstrain caused by an inhomogeneous distribution of intercalates in the active material. In addition, two new peaks of unclear origin emerge in the highly degraded KCl sample, indicating further structural changes. More systematic operando characterizations monitoring the structure evolution of CuHCF during cycling will be needed to obtain more detailed insights into the fading mechanisms.

SEM analysis of the cathode after cycling in 4 mol kg^⁻1^ KCl reveals significant morphological changes to the CuHCF nanosheets (Figure S5D, Supporting Information). While the overall shape of the particles remains unaltered, etched holes and pores can be observed on the active material, suggesting severe transition metal dissolution consistent with the electrochemical results. In contrast, as shown in Figure S5B (Supporting Information), this effect is not seen after cycling the cathode in 4 mol kg^⁻1^ MgCl_2_, indicating that the material dissolution is suppressed in this electrolyte.

Based on these findings, a stabilization mechanism in the aqueous Mg‐ion electrolyte can be proposed. Prior studies have shown that the capacity fading of PBAs is closely associated with water incorporation into the structure and the interaction between free water in the electrolyte and the active material.^[^ ^47^, ^53^, ^54^ ^]^ Hydrolysis and specific adsorption of hydroxide ions (OH⁻) are known to promote transition metal dissolution, ultimately leading to structural degradation and capacity loss. Therefore, the interaction between cations and water plays a critical role in the electrochemical stability of PBA electrodes. Multivalent ions, due to their higher charge density, form larger hydration shells and exhibit stronger interactions with water molecules and OH⁻ anions compared to monovalent ions of similar ionic radius. As a result, water molecules and hydroxide species are more likely to coordinate with the electrolyte cations rather than interact with the PBA framework. This reduces the extent of hydrolytic degradation at the electrode surface. Experimental evidence from the XRD and SEM analyses supports this mechanism. In the KCl electrolyte, capacity fading correlates with pronounced structural changes and active material dissolution. The XRD patterns show the disappearance of characteristic peaks after cycling, along with the emergence of new peaks indicative of phase transformation. SEM images further reveal significant morphological degradation and material loss. In contrast, these changes are absent in the MgCl_2_‐based electrolyte. This suggests that the lower concentration of free water and reactive OH⁻ species in the MgCl_2_ electrolyte effectively suppresses the degradation process, thereby enhancing electrode stability.

In summary, CuHCF displays the highest and, thus, most favorable intercalation potential in the presence of K‐ions. However, the cycling stability in the 4 mol kg^⁻1^ KCl electrolyte is insufficient and can be largely improved by using divalent‐ion‐based electrolytes such as MgCl_2_ and CaCl_2_. As the stabilization is linked to the interaction between the divalent cations and the aqueous environment, it should also be achieved when using, e.g., MgCl_2_ as an electrolyte additive for KCl. Hence, the performance of the cathode was further investigated in a mixed 1.8 mol kg⁻^1^ KCl + 1.8 mol kg^⁻1^ MgCl_2_ aqueous electrolyte. Figure 2B shows a representative charge and discharge cycle of CuHCF in 4 mol kg^⁻1^ KCl, 4 mol kg^⁻1^ m MgCl_2_, and the mixed electrolyte. In the Mg‐based electrolyte, the plateaus during intercalation and deintercalation are significantly more sloped than in 4 mol kg^⁻1^ KCl. Furthermore, as already discussed above, the average discharge potential in MgCl_2_ is lower and thus less favorable. However, when using a bi‐salt electrolyte containing both K^+^ and Mg^2+^, the profile closely resembles the pure KCl electrolyte, indicating that the reduction is facilitated by the presence of K^+^. That is further confirmed when analyzing the CVs of CuHCF in the three aforementioned electrolytes. As shown in Figure 2C, the peak positions and shapes in the mixed electrolyte closely match the curve for 4 mol kg^⁻1^ KCl. In 4 mol kg^−1^ MgCl_2_, however, the cathode displays significantly broader peaks and a higher peak‐to‐peak separation due to the more sluggish charge transfer and diffusion kinetics of the Mg^2+^ ions. That implies that adding MgCl_2_ to the K‐based electrolyte does not significantly influence the charge transfer kinetics and electrode potential. As K^+^ features a much smaller hydration shell, its intercalation should be favored over the intercalation of Mg^2+^. However, energy‐dispersive X‐ray spectrometry (EDX) analysis shows the presence of both K and Mg in a CuHCF cathode reduced in the mixed electrolyte (Figure S6, Supporting Information). Hence, although the ion transfer kinetics are the same as in the pure KCl electrolyte, both Mg^2+^ and K^+^ intercalate into the electrode in the mixed electrolyte, since the reduction and oxidation peaks, representing their respective (de‐)intercalation potentials, overlap. Therefore, the mixed electrolyte is capable of combining the beneficial properties of both pure electrolytes. While the electrode potential itself is not affected by the additional Mg‐ions, the electrolyte modification improves other important electrode properties. At 1 C, the coulombic efficiency is increased from ≈95% to ≈99% compared to the pure KCl electrolyte due to the water‐binding effect of Mg^2+^ and the acidification of the electrolyte by the Lewis acid.

Figure 2D displays the capacity retention of the CuHCF electrode over 100 cycles at 10 C in the single salt and mixed electrolytes. As discussed above, the cycling stability is largely improved when Mg^2+^ is intercalated instead of K^+^. A similar effect is observed in the mixed electrolyte as the capacity retention increases from ≈40% in KCl to over 90% in the bi‐salt electrolyte. This proves that the stabilization through water‐ and OH‐capturing can be achieved by using an Mg salt as an additive to an alkali‐ion‐based electrolyte. As evidenced by the EDX results, both Mg and K intercalate into the PBA structure. Therefore, the effects of Mg ions on interstitial water and OH^−^ species also occur in the mixed electrolyte, and the same stabilization mechanism as in the pure MgCl_2_ electrolyte can be expected. Using mixed electrolytes enables a longer cycle life for CuHCF while maintaining the flat discharge profile and high electrode potential associated with the K‐ion‐based electrolyte.

To evaluate the performance of the cathode in the 1.8 mol kg^⁻1^ KCl + 1.8 mol kg^⁻1^ MgCl_2_ mixed electrolyte in more detail, galvanostatic cycling was carried out at different rates ranging from 1 C (65 mA g^−1^) to 300 C (19.5 A g^−1^). Figure 2E shows the specific capacity and coulombic efficiency for each cycle. At 1 C, the electrode displays a maximum discharge capacity of 65 mAh g^−1^, of which ≈85% can be retained at 10 C and ≈40% can be retained at an ultrahigh rate of 300 C. The maximum specific capacity exceeds the values commonly reported for CuHCF cathodes prepared by the conventional coprecipitation method, which can be linked to the low vacancy and water content and the change in crystal structure achieved by the synthesis procedure using sodium pyrophosphate as a chelating agent.^[^ ^18^, ^41^ ^]^ The high crystallinity and large interstitial sites further facilitate fast ion transport kinetics, enabling a relatively high rate capability. The reaction kinetics were further analyzed by recording CVs at scan rates between 1 and 50 mV s^−1^. The peak current *i*_peak_ can be related to the scan rate *ν* by the empirical formula *i*_peak_ *= b∙ν^a^* with the parameter *a* indicating the rate‐limiting process. For purely mass‐transport‐limited kinetics, as commonly observed in intercalation‐type electrode materials *a* approaches 0.5. For purely surface‐limited kinetics, such as for capacitive charge storage in supercapacitors, *a* approaches 1.0. As shown in Figure 2F, the parameter *a* lies at 0.59 for CuHCF in the mixed electrolyte, indicating mostly mass transport limited kinetics.

**Figure 3:**
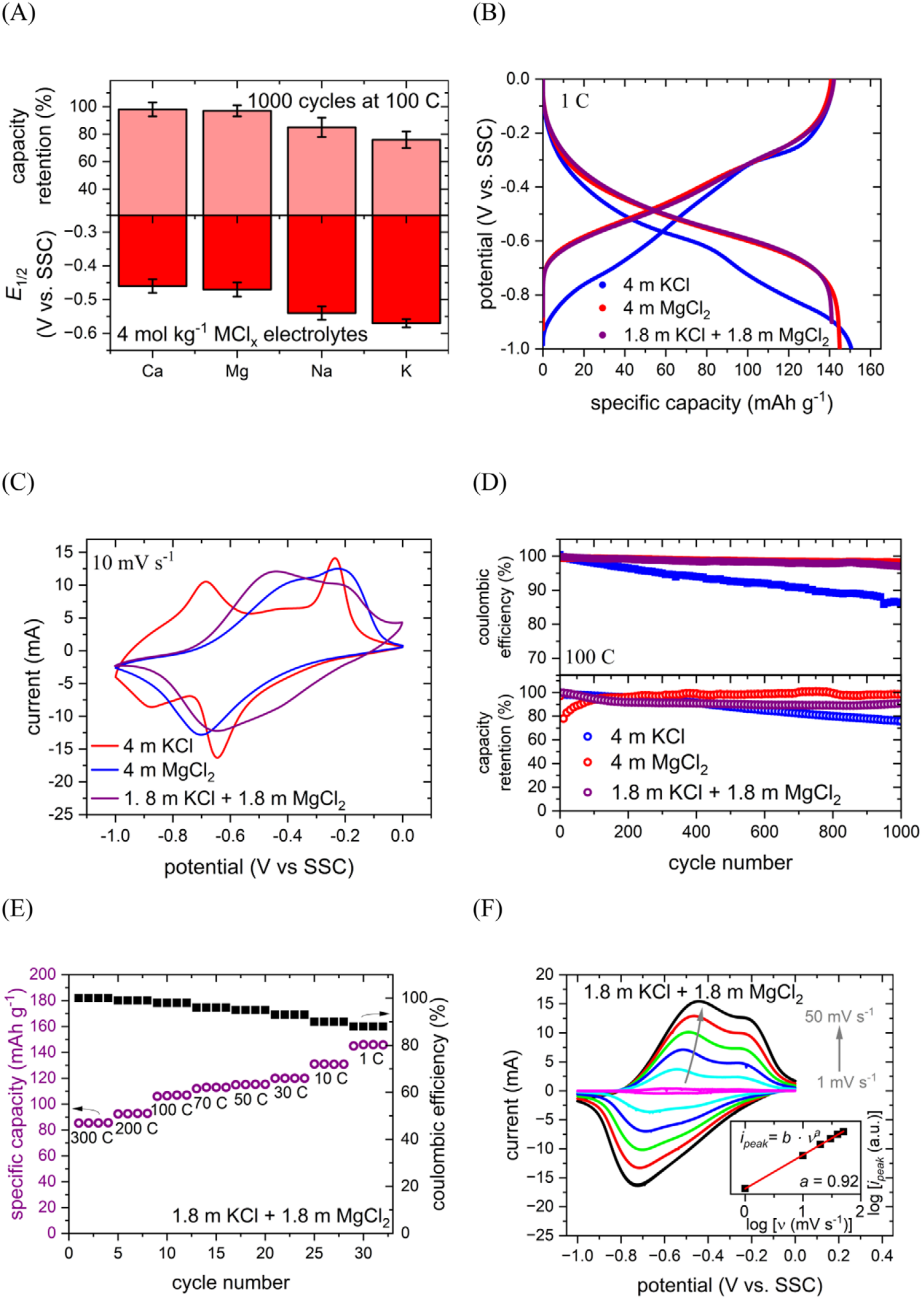
Electrochemical performance of PNFE. A) Summary of the electrode potential and cycling stability in different 4 mol kg^−1^ MCl*_x_* (M = Na, K, Mg, Ca) aqueous electrolytes. Data presented as mean ± SD, *n =* 5. B) Galvanostatic (dis‐)charge curves, C) CVs, and D) cycling stability in KCl, MgCl_2_, and 1.8 mol kg^⁻1^ KCl + 1.8 mol kg^⁻1^ MgCl_2_. E) Specific discharge capacity in 1.8 mol kg^⁻1^ KCl + 1.8 mol kg^⁻1^ m MgCl_2_. F) CVs at different scan rates in 1.8 mol kg^⁻1^ KCl + 1.8 mol kg^⁻1^ MgCl_2_.

CV and galvanostatic cycling with potential limitations (GCPL) measurements were carried out on PNFE to investigate the influence of the electrolyte composition on the electrochemical performance of the anode. **Figure** **3**A shows an overview of the cation effects on the most important electrode properties. Similarly to the cathode, significant changes in electrode potential and stability can be observed when varying the cationic species in the electrolyte. PNFE displays the most negative and, thus, most favorable half‐charge potential in the presence of K^+^. As this electrolyte also enables the highest cathode potential, a K‐based battery should deliver the highest cell voltage and energy density among the investigated systems. Comparing all cations, the anode potential follows the K^+^ < Na^+^ < Mg^2+^/Ca^2+^ trend with no discernible difference between Ca^2+^ and Mg^2+^. As the charge storage mechanism of PNFE does not feature intercalation into a crystal lattice, the hydration energy of the cation does not seem to play a role in determining the redox potential. However, it is closely linked with the charge density of the inserted ion. The high charge density of Mg^2+^ and Ca^2+^ facilitates the enolization process, yielding more positive potentials, while the low charge density of the monovalent Na^+^ and K^+^ results in more negative potentials.

As for the cathode, the electrolyte composition also affects the cycling stability of the anode. Since PNFE exhibits high fast‐charging capabilities, the electrode was cycled at a C‐rate of 100 C for 1000 cycles to assess the degradation. Generally, only small capacity losses can be observed in all electrolytes, but they are slightly higher in solutions based on K^+^ and Na^+^. This capacity fading is most likely related to material detachment from the electrode, as there are no known degradation mechanisms for polyimides. To confirm this hypothesis, SEM images of the PNFE anode were taken before and after cycling in the KCl electrolyte. As shown in Figure S7 (Supporting Information), the anode displays significantly enlarged cracks on the surface and detached flakes in multiple areas. Furthermore, the detached solid material was observed floating in the electrolyte at the bottom of the cell. Due to the larger cation hydration shell and higher anion concentration, solutions of MgCl_2_ and CaCl_2_ are more viscous than KCl and NaCl at the same concentration (Figure S4, Supporting Information), inhibiting the transport of dissolution products away from the electrode. However, it should be noted that for all electrolytes, this effect would be less pronounced in realistic cell setups containing significantly smaller volumes of electrolyte. Hence, it can be assumed that the cycle life of the full battery using the two mentioned electrode materials will mostly depend on the cathode stability.

A 1.8 mol kg^⁻1^ KCl + 1.8 mol kg^⁻1^ MgCl_2_ was identified to yield optimal cycling stability and electrode potential for the CuHCF cathode. Therefore, the electrochemical performance of PNFE was also analyzed in this electrolyte. Figure 3B shows the charge and discharge curves of the anode in pure KCl, pure MgCl_2_, and the mixed electrolyte recorded at 1 C. The (dis‐)charge profiles for the Mg‐based and the bi‐salt electrolytes are almost identical, indicating that Mg^+^ insertion is preferred over K^+^. Although the lower average reduction and oxidation potentials in the presence of K^+^ are favorable for achieving higher full‐cell voltages, the plateaus are less sloped in the presence of Mg^2+^. As shown in Figure 3C, the CV of PNFE in KCl displays a significant potential difference between the first and second electron transfer and a large peak‐to‐peak separation for the first redox step. In contrast, the two reduction and oxidation peaks overlap in the Mg‐based and mixed electrolytes. As demonstrated in our previous work, the underlying charge transfer mechanism of PNFE does not change when divalent ions are inserted instead of monovalent ions.^[^ ^46^ ^]^ However, as multiple polymer units are involved in the storage of a single Mg^2+^ ion, the oxidation and reduction peaks can no longer be clearly distinguished since interactions between the redox active centers lead to further peak broadening and overlapping. When using a bi‐salt electrolyte, the general CV shape is not affected, but the peak‐to‐peak separation decreases due to the enhanced electrolyte conductivity enabled by the higher mobility of K^+^. Figure 3D shows the capacity retention of PNFE per cycle in 4 mol kg^⁻1^ KCl, 4 mol kg^⁻1^ MgCl_2_, and 1.8 mol kg^⁻1^ KCl + 1.8 mol kg^⁻1^ MgCl_2_ recorded at 100 C over 1000 cycles. Although relatively slow, a continuous capacity decay accompanied by a decrease in coulombic efficiency can be observed in the pure KCl electrolyte. In MgCl_2_ and the bi‐salt electrolyte, the anode remains stable after the initial cycles, displaying no significant capacity fading and high coulombic efficiencies. The capacity growth occurring during the first cycles in pure MgCl_2_ can be linked to delayed electrode wetting due to the high viscosity of this electrolyte.

To investigate the electrochemical performance of the PNFE anode in the mixed electrolyte, GCPL, and CV measurements were carried out at different C‐rates and scan rates, respectively. In Figure 3E, the specific capacities per cycle for different charging and discharging rates ranging from 1 C (150 mA g^−1^) to 300 C (45 A g^−1^) are displayed. At 1 C, the anode exhibits a maximum discharge capacity of 148 mAh g^−1^, of which ≈85% are retained at 10 C and ≈60% are retained at 300 C. While the coulombic efficiency decreases slightly at lower rates due to parasitic HER, this effect is more pronounced in the flooded cell setup compared to a realistic battery assembly with minimal electrolyte volume. To understand the exceptional rate capability of PNFE, the peak current of CVs recorded at different scan rates is evaluated in Figure 3F. For the anode, the value of parameter *a*, introduced previously when studying the cathode, lies at 0.92, indicating that the kinetics are mostly surface‐limited. This reflects the pseudo‐capacitive nature of the charge storage mechanism of PNFE, which translates to fast charge transfer kinetics.

**Figure 4:**
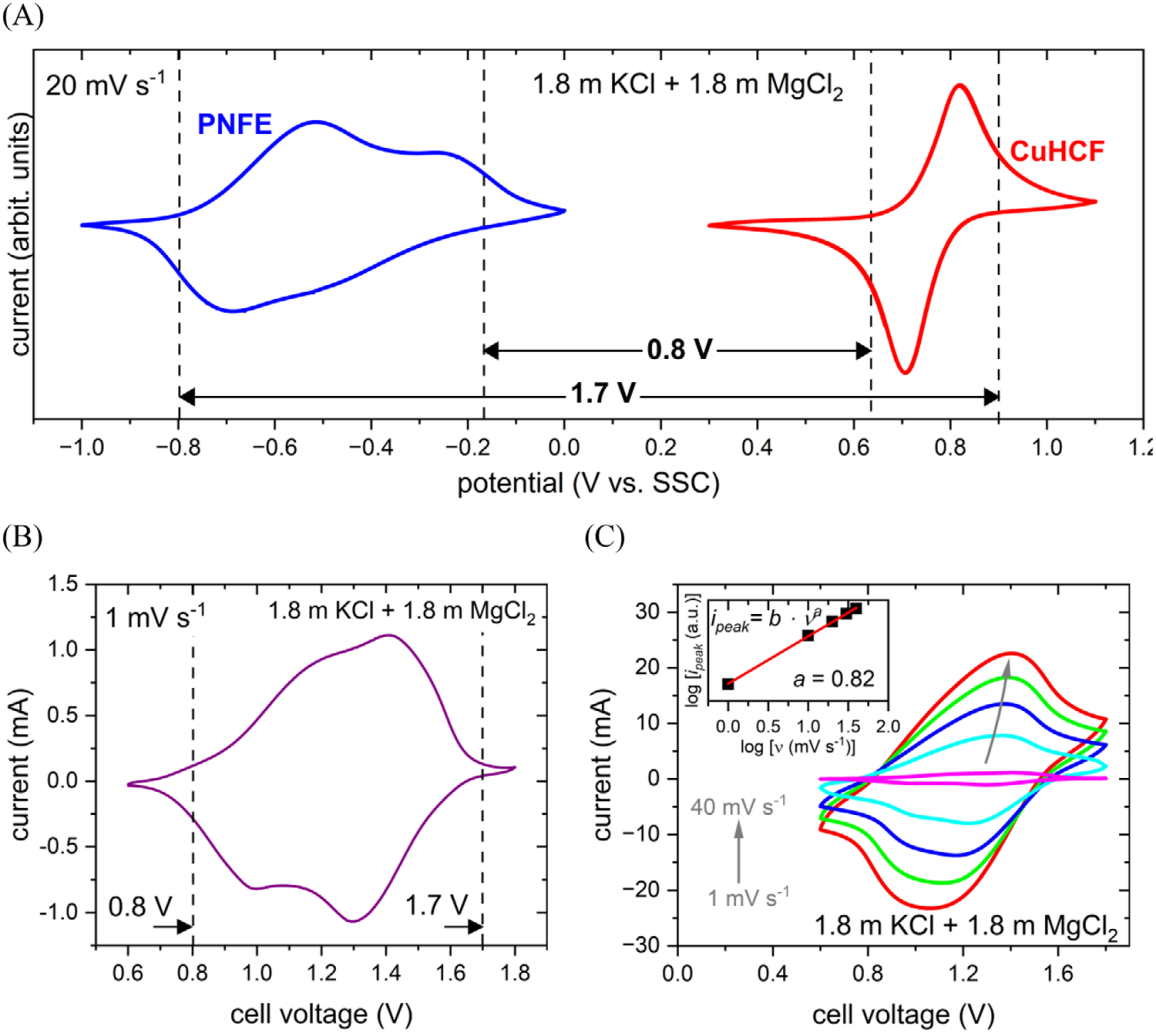
CV analysis of the PNFE/1.8 mol kg^⁻1^ KCl + 1.8 mol kg^⁻1^ MgCl_2_/CuHCF full cell. A) CVs of anode and cathode in the bi‐salt electrolyte. B) CV of the full cell at 1 mV s^−1^. C) CVs of the full cell at different scan rates. Inset: peak current versus scan rate.

Both electrodes exhibited promising electrochemical performance in the mixed electrolyte and were thus combined into a model aqueous battery. **Figure** **4**A shows the combined CV of the PNFE anode and the CuHCF cathode in the bi‐salt electrolyte.

Considering the redox‐active potential regions of the single electrodes, the battery should be able to operate at cell voltages between 0.8 and 1.7 V. Furthermore, no parasitic HER or OER currents can be observed in the CVs, indicating that the electrolyte stability window is sufficient for this material combination. A CV of the full cell recorded at 1 mV s^−1^ is depicted in Figure 4B, confirming that the battery displays highly reversible charge and discharge peaks within the expected voltage range. Two oxidation peaks at 1.17 and 1.42 V and two reduction peaks at 1.01 and 1.30 V are recorded, reflecting the behavior of the anode and cathode. To analyze the charge transfer kinetics of the full cell, CVs were recorded at different scan rates ranging from 1 to 40 mV s^−1^ (Figure 4C). For the full cell, parameter *a*, extracted from the dependence of the peak current on the scan rate, yields 0.82, lying between the anode and cathode values and indicating a partially mass‐transport‐limited process. As the anode exhibits almost no diffusion limitations, the kinetics of the full cell are probably limited by the cathode. Hence, the preferential intercalation of K^+^ over Mg^2+^ into CuHCF enables enhanced rate capabilities as divalent ions feature higher charge densities and, thus, interact more strongly with the host lattice. This allows for the control of the fast‐charging capability of the full cell by tuning the capacity ratio of the electrodes since an oversized cathode will improve the high‐rate performance. In contrast, balanced cathode and anode capacities enable higher energy densities at low rates. For this study, a ratio between anode and cathode active material of 1:2 was chosen to achieve nearly equal electrode capacities.

**Figure 5:**
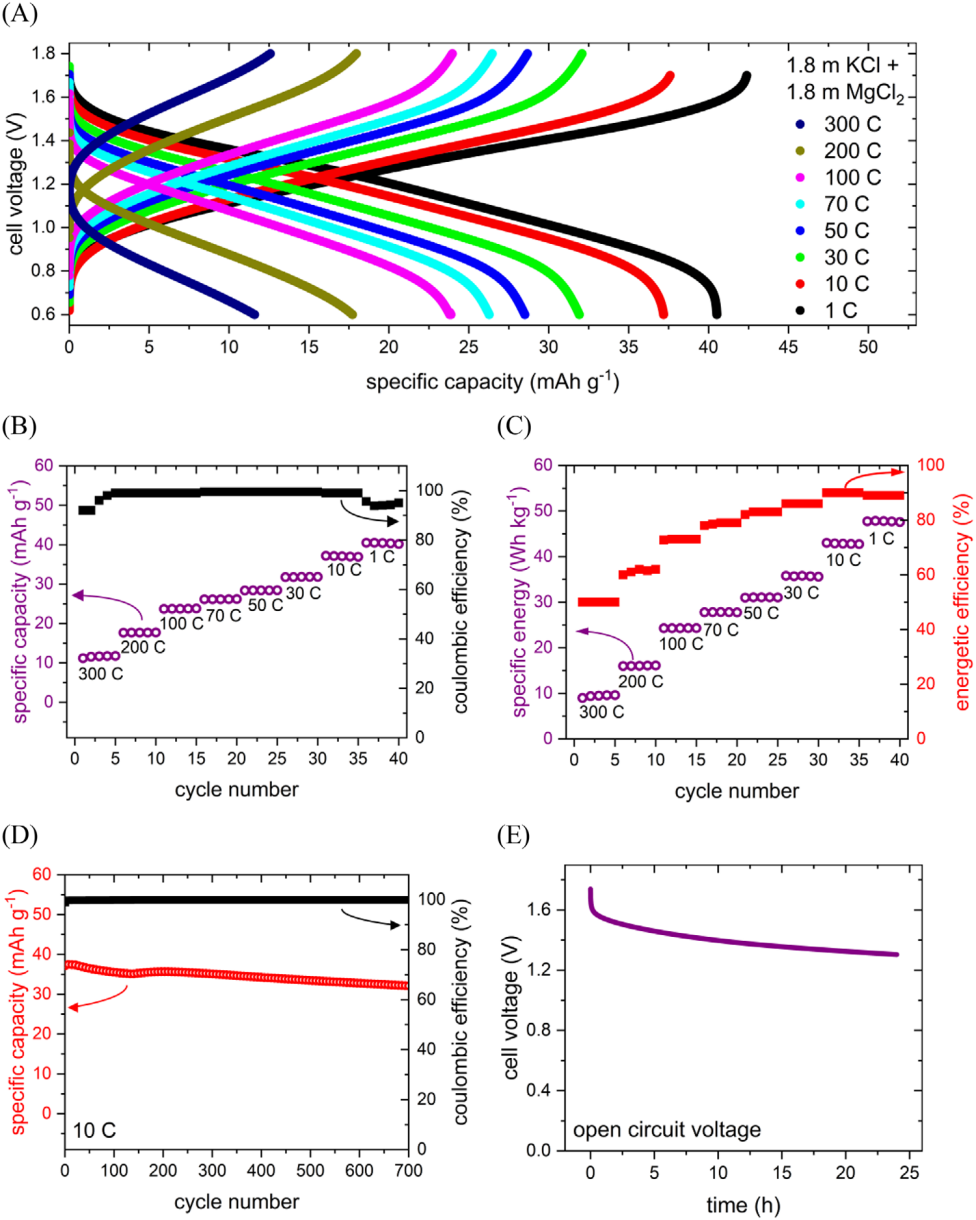
Electrochemical performance of the PNFE/1.8 mol kg⁻^‐^ KCl + 1.8 mol kg^⁻1^ MgCl_2_/CuHCF full cell. A) (Dis‐)charge curves of the full cell at different current densities. B) Specific capacity and C) specific energy for each cycle during a rate capability test. D) Cycling stability. (E) Open‐circuit voltage. (Energy densities are normalized to the combined anode and cathode active material mass).

**Figure** **5**A shows the charge and discharge profiles of the CuHCF/1.8 mol kg^⁻1^ KCl + 1.8 mol kg^⁻1^ MgCl_2_/PNFE aqueous battery recorded at C‐rates between 1 C (42 mAh g^−1^) and 300 C (12.6 A g^−1^). To ensure stable operating conditions and monitor any rate‐dependent degradation, multiple cycles were recorded at each C‐rate. At a discharge rate of 1 C, the voltage curve features a sloping plateau between 1.6 and 0.8 V, with an average discharge voltage of 1.22 V. Within the 0.6–1.7 V range, the cell achieves a reversible capacity of 42 mAh g^−1^, corresponding to ≈90% of the theoretical value calculated from the specific capacities of the anode (148 mAh g^−1^) and cathode (65 mAh g^−1^). The battery demonstrates strong fast‐charging capability, retaining ≈90% of its capacity at 10 C, 57% at 100 C, and 30% at 300 C. This can be attributed to the low charge density and hydration energy of K^+^ enabling fast diffusion kinetics at the cathode, which can be considered the rate‐limiting process within the battery. As shown in Figure 5B, coulombic efficiencies exceed 95% across all C‐rates, affirming the cell's high reversibility.

By integrating the discharge profile, the specific discharge energy for each cycle and C‐rate is evaluated with regard to the combined active material mass of the anode and cathode and displayed in Figure 5C. Due to increased voltage polarization at higher rates, the specific energy shows a stronger dependence on the rate than the specific capacity. However, the specific energy declines only slightly from 48 Wh kg^−1^ at 1 C to 43 Wh kg^−1^ at 10 C, indicating the battery can achieve fast‐charging times of 5–10 min with minimal energy loss. At 10 C, the cell also demonstrates its highest energetic efficiency of 90%. While voltage polarization reduces efficiency at higher rates, coulombic losses from parasitic HER and OER contribute to minor efficiency decreases at lower rates. It should be noted that besides OER, Cl‐oxidation can contribute to parasitic currents at the cathode in electrolytes containing Cl^−^‐anions. However, due to its significantly higher onset potential, this reaction most likely plays a minor role compared to the OER. Consequently, this cell is suitable for applications requiring moderate energy densities but fast‐charging capabilities, such as public transportation or grid stabilization, and can even operate effectively at high rates of 100, 200, and 300 C. To further support this conclusion, the power density can be calculated by multiplying the specific discharge current by the average discharge voltage at each rate. The specific currents span from 42 mAh g^−1^ (1 C) to 12.6 A g^−1^ (300 C), while the average discharge voltages range from 1.22 V (1 C) to 0.85 V (300 C). This results in power densities between 51.2 W kg^−1^ and 10.7 kW kg^−1^, representing some of the highest values reported for aqueous batteries to date.

To assess the cycling stability, the CuHCF/1.8 mol kg^⁻1^ KCl + 1.8 mol kg^⁻1^ MgCl_2_/PNFE aqueous battery was charged and discharged repeatedly at 10 C. Figure 5D shows the specific capacity and coulombic efficiency for each cycle. The cell exhibits good cycle performance, retaining ≈90% of its initial capacity after 700 cycles while also maintaining high coulombic efficiency of nearly 100% throughout the entire measurement. To confirm the successful suppression of water‐splitting side reactions in the battery based on the bi‐salt electrolyte, the cell was initially charged to a voltage of 1.7 V, and then the cell voltage was recorded for 24 h under open‐circuit conditions (Figure 5E). After an initial drop to ≈1.5 V, the open‐circuit voltage (OCV) remains relatively stable, indicating that the cell exhibits no significant self‐discharge.

To further demonstrate the superior properties of the aqueous battery based on the bi‐salt electrolyte, multiple cells using the same electrode materials were assembled with various electrolytes. Subsequently, the average discharge voltage and cycling stability were assessed for at least five cells for each combination.

**Figure 6:**
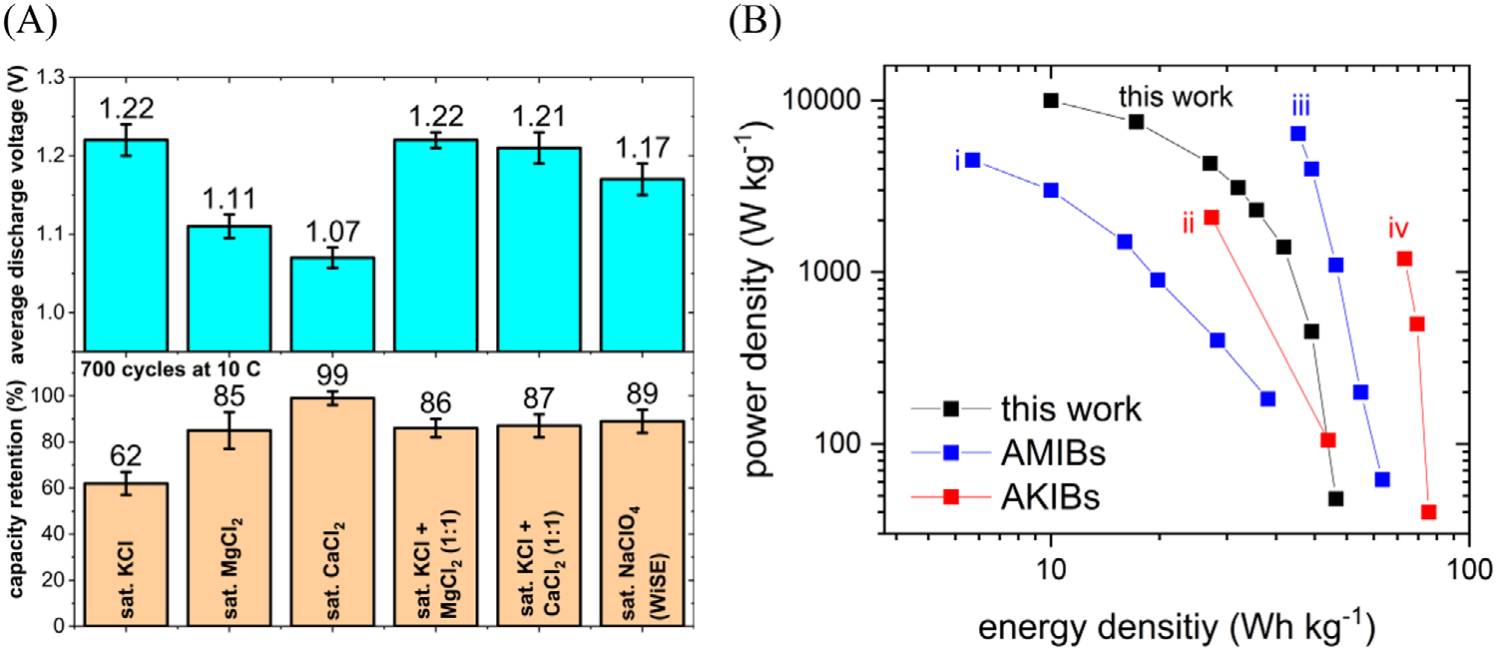
A) Comparison of the cell voltage and cycling stability of PNFE//CuHCF batteries with different electrolytes. Data presented as mean ± SD, *n =* 5. B) Comparison of the aqueous battery performance in this work with AKIBs and AMIBs reported by i) Chen et al.^[^ ^45^ ^]^ ii) Wang et al.,^[^ ^61^ ^]^ iii) Wang et al.,^[^ ^62^ ^]^ and Jiang et al.^[^ ^25^ ^]^

An overview of the results is given in **Figure** **6**A. As expected from the single‐electrode measurements, the battery featuring saturated (4.6 mol kg^−1^) KCl suffers from significant capacity fading while providing a relatively high cell voltage. In contrast, the cells using electrolytes based on divalent Ca^2+^ and Mg^2+^ deliver substantially lower cell voltages but show improved capacity retention. Especially when using saturated (6.7 mol kg^−1^) CaCl_2,_ the battery exhibits almost no capacity loss over 700 cycles due to the high concentration and viscosity of this electrolyte. For a more detailed analysis of this highly stable configuration, the reader is referred to our previous work.^[^ ^37^ ^]^ However, as higher cell voltages are desirable for maximizing energy density, the use of mixed electrolytes containing K‐ions together with divalent species proves to be a promising strategy for optimizing battery performance. Both the cells based on 1.8  KCl + 1.8 mol kg^−1^ MgCl_2_ and 2 mol kg^−1^ KCl + 2 mol kg^−1^ CaCl_2_ show better capacity retention than the K‐ion battery and higher cell voltages than the Mg‐ and Ca‐ion systems. In addition, a reference cell containing a commonly used NaClO_4_ water‐in‐salt electrolyte displays similar capacity retention and a slightly lower cell voltage, confirming that the bi‐salt electrolyte approach yields competitive results compared to the more expensive WiSE strategy. To confirm this conclusion, a comparison between the optimized battery proposed in this work and state‐of‐the‐art aqueous K‐ion (AKIB) and Mg‐ion batteries (AMIB) reported in the literature is shown in Figure 6B. Whereas the batteries developed by Wang et al. and Jiang et al. exhibit higher energy densities, they rely on fluorinated Mg(TFSI)_2_ and KCF_3_SO_3_ salts, respectively.^[^ ^25^, ^62^ ^]^ It should be noted that while Jian et al. work with very high concentrations to enable stable cycling of the Mn‐PBA cathode, Wang et al. use a relatively dilute electrolyte. However, the used Li_3_V_2_(PO_4_)_3_ cathode material features both lithium and vanadium, raising the expected cost of this configuration. Nevertheless, the superior energy densities of these systems render them suitable for applications requiring lighter cell weight. The energy density of the cell in our work exceeds the values typically reported for aqueous batteries based on F‐free dilute electrolytes, such as the AKIB and AMIB reported by Chen et al. and Wang et al., respectively.^[^ ^45^, ^61^ ^]^ In addition, power densities of more than 10 kW kg^−1^ are superior to all of the designs mentioned above and have, to the best of our knowledge, rarely been reported for aqueous batteries so far.

## 3 Conclusions

This study demonstrates that the choice of cation strongly influences key electrode properties, such as redox potential and cycling stability. Among the cations examined, potassium ions generally provide the highest electrode potential at the CuHCF cathode, but the cycling stability in a 4 mol kg^−1^ KCl electrolyte remains inadequate. At the same concentration, divalent cations like Mg^2^⁺ and Ca^2^⁺ significantly improve cycling performance, albeit at the cost of reducing the intercalation potential. However, we show that combining monovalent K⁺ with divalent ions optimizes both stability and electrode potential. Similar trends were observed for the PNFE anode, where K⁺ results in the lowest insertion potential, while Ca^2^⁺ and Mg^2^⁺ lead to the longest cycle life.

Building on these insights, we developed a full aqueous battery using the specified electrode materials and a 1.8 mol kg^−1^ KCl + 1.8 mol kg^−1^ MgCl_2_. This battery achieves a competitive energy density of 48 Wh kg^−1^ at 1 C, with an energy efficiency of 95%, and a power density exceeding 10 kW kg^−1^ at 300 C. It also demonstrates excellent fast‐charging capabilities, retaining 70% of its capacity at a high rate of 50 C, making it suitable for supercapacitor‐like applications, such as applications in the public transport sector. By utilizing abundant, low‐cost salts in a safe and environmentally friendly electrolyte, we believe that our work represents a significant step toward the practical application of aqueous beyond‐lithium‐ion batteries.

## 4 Experimental Section

### Material Synthesis

All chemicals were purchased from commercial suppliers and used without further purification.

PNFE was synthesized for a previous work and used without further processing. For detailed synthesis information, the reader is referred to a previous publication.^[^ ^46^ ^]^ In short, 10 mmol of 1,4,5,8‐naphthalene tetracarboxylic dianhydride (NTCDA) and 10 mmol of ethylenediamine (EDA) were dissolved in 20 mL of degassed N‐methyl pyrrolidone (NMP) in a Schlenk flask. The mixture was heated to 215 °C in a sand bath and refluxed overnight. The resulting dark precipitate was filtered and washed several times with NMP and water. It was then dried under vacuum at 0.08 mbar, progressively increasing the temperature from room temperature to 120 °C and finally to 210 °C. The final product was a gray‐brown powder with a high yield of 92% (2.7 g).

*Elemental Analysis of PNFE*: Calcd. for an infinite polymer (C_16_H_8_N_2_O_4_), %: C 65.76, H 2.76, N 9.59; calcd. for an octamer, %: C 65.10, H 3.03, N 10.52; found, %: C 65.13, H 2.91, N 10.32.

Copper hexacyanoferrate (CuHCF) was prepared by coprecipitation from two solutions according to the method reported by Xu et al.^[^ ^41^ ^]^ To prepare solution A, 1 mmol of CuCl_2_ ∙ 2 H_2_O and 1 mmol of Na_4_P_2_O_7_ ∙ 10 H_2_O were dissolved in 20 mL of deionized (DI) water. For solution B, 1 mmol of Na_4_Fe(CN)_6_ ∙ 10 H_2_O, 0.3 g of polyvinylpyrrolidone (PVP), and 0.58 g of NaCl were mixed into 50 mL of DI water. Solution A was then gradually added to solution B, while vigorously stirring at room temperature. The mixture was left to age for 5 d, after which the precipitate was separated by centrifugation and washed three times each with water and ethanol. Finally, the product was dried overnight at 70 °C.

### Electrode Preparation

As reported in a previous work, PNFE and CuHCF electrodes were fabricated using the slurry casting method.^[^ ^37^ ^]^ For the PNFE slurry, a mixture consisting of 80 wt% polyimide as the active material, 10 wt% carbon black super C‐65 (MTI, USA), and 10 wt% Nafion binder (5 wt% in lower aliphatic alcohols and water, Sigma Aldrich, USA) was ground using a planetary ball mill. Water and isopropyl alcohol in a 1:1 ratio were added as dispersing agents, and the mixture was stirred vigorously to form the anode slurry.

The CuHCF slurry was prepared similarly, using 80 wt% active material, 10 wt% carbon black super C‐65, and 10 wt% polyvinylidene fluoride (PVDF, MTI, USA), also ground in a planetary ball mill. NMP was added during stirring to create the cathode slurry.

Both slurries were cast onto pyrolytic graphite sheet current collectors. The mass loading of the active material was ≈3.5 mg cm⁻^2^ for the CuHCF cathode and 2.0 mg cm⁻^2^ for the PNFE anode.

### Material Characterization

The PNFE anode was characterized by field emission SEM (JSM‐7500F, JEOL). FTIR (Bruker Vertex‐70) spectroscopy and XPS (Al Kα source, SPECS XR50 Xf‐Ray, SPECS PHOIBOS 150 hemispherical analyzer, SPECS spectrometer). SEM and powder XRD (Rigaku MiniFlex 600‐C) were used to analyze the structure of the CuHCF cathode.

The viscosities of the electrolytes were measured at 25 °C with a commercial shear rheometer (MCR 302, Anton Paar GmbH, Graz, Austria) equipped with a cone‐shaped measuring head (CP50‐1, 79040, Anton Paar) and an air‐cooled bottom plate (P‐PTD 200/56/Air, Anton Paar).

Scanning electron microscopy – energy‐dispersive X‐ray spectrometry (SEM‐EDX, JSM‐IT200, JEOL) was conducted to analyze the elemental composition of CuHCF after being reduced in the mixed electrolyte.

Nitrogen gas sorption isotherms were measured using a Micromeritics 3Flex analyzer under liquid nitrogen at 77 K to determine surface area and pore size distribution. The BET specific surface area and pore size distribution were analyzed with the dedicated software provided by Micromeritics. Prior to measurement, the prepared electrodes and bare substrate were cut into small pieces, placed in a tubular glass measuring cells, and activated under vacuum at 120 °C for 16 h to remove any residual moisture or contaminants.

### Electrochemical Measurements

Single‐electrode measurements were performed using a three‐electrode setup within a sealed glass cell, maintained under a constant argon flow to ensure an inert atmosphere. Both the electrolyte and the glass cell were purged with argon for at least 10 minutes before introducing the electrolyte into the cell. A platinum wire (MaTeck, Germany) served as the counter electrode, while an Ag/AgCl electrode (SSC, 3 m KCl, SI Analytics), connected to the electrolyte through a Luggin capillary, was used as the reference electrode. 4 mol kg^−1^ (4 m) NaCl, KCl, MgCl_2_, and CaCl_2_ were used as single‐salt electrolytes. A saturated mixture of 1.8 mol kg^−1^ KCl and 1.8 mol kg^−1^ MgCl_2_ was prepared as a bi‐salt electrolyte.

A BioLogic VSP‐300 potentiostat was used to conduct CV and GCPL. CVs were recorded at different scan rates ranging from 1 to 50 mV s^−1^ at potentials from −1.0 to 0.0 V and from 0.3 to 1.1 V (vs SSC) for anode and cathode, respectively. Galvanostatic (dis‐)charging was carried out at C‐rates between 1 and 300 C within the same potential ranges. By galvanostatically charging and discharging the electrodes for 100 cycles at 10 C (cathode) and for 1000 cycles at 100 C (anode), the cycling stability in different electrolytes was tested.

### Full‐Cell Assembly and Measurements

To address the corrosive effects of chlorides on most stainless steel alloys, a 3D‐printed plastic cell with pyrolytic graphite foil as contacts was used for the full‐cell assembly. The aqueous bi‐salt battery was constructed in a nitrogen‐filled glovebox to prevent oxygen from dissolving into the electrolyte. A commercial alkaline battery separator, pre‐soaked in a 1.8 mol kg^−1^ KCl + 1.8 mol kg^−1^ MgCl_2_ electrolyte, was placed between the electrodes. The cell was fully sealed with rubber O‐rings to ensure a closed environment.

For the full‐cell, CV, GCPL, and OCV measurements were performed using a Biologic VSP‐300 potentiostat. CVs were measured at scan rates ranging from 1 to 40 mV s⁻¹, with cell voltages between 0.6 and 1.8 V. GCPL was carried out at C‐rates from 300 to 30 C over the same voltage range. For the 10 and 1 C rates, the upper voltage limit was slightly reduced to 1.7 V to prevent side reactions. The OCV was monitored for 24 h after fully charging the battery with a constant voltage hold at 1.7 V.

### Statistical Analysis

All statistical values are expressed as mean ± SD (standard deviation) with the number of samples *n* given in the figure captions. Statistical analysis was carried out using Origin 2023 software.

## Conflict of Interest

The authors declare no conflict of interest.

## Author Contributions

R.S. and A.B. proposed the concept and co‐wrote the manuscript. R.S., S.R., S.W., J.S., P.S., and D.G. performed the experiments. R.S., A.S., J.S., and M.H. analyzed the data. All authors contributed to data analysis and manuscript conceptualization.

## Supporting information



Supporting Information

## Data Availability

The data that support the findings of this study are available from the corresponding author upon reasonable request.
