# Supplementary material for: Sustainable High‐Performance Aqueous Batteries Enabled by Optimizing Electrolyte Composition
Source: Adv Sci (Weinh). 2025 May 5;12(25):2417587. doi: 10.1002/advs.202417587 (PMC12224927; doi:10.1002/advs.202417587)
Supplement: Supplementary file 1 — Supporting Information [file ADVS-12-2417587-s001.docx]

**Supporting Information for:**

**Sustainable High-Performance Aqueous Batteries Enabled by Optimizing Electrolyte Composition**

*Raphael L. Streng, Samuel Reiser, Anatoliy Senyshyn, Sabrina Wager, Johannes Sterzinger, Peter Schneider, David Gryc, Mian Zahid Hussain, Aliaksandr S. Bandarenka**

R. L. Streng, S. Reiser, S. Wager, J. Sterzinger, P. Schneider

Physics of Energy Conversion and Storage, Department of Physics, Technische Universität München (TUM), James-Franck-Str. 1, 85748 Garching, Germany.

A. Senyshyn
Heinz Maier-Leibnitz Zentrum (MLZ), Technische Universität München, Lichtenbergstr. 1, Garching, Germany.

D. Gryc, M. Z. Hussain

Chair of Inorganic and Metal-Organic Chemistry, Department of Chemistry, School of Natural Sciences, TUM, Lichtenbergstraße 4, 85748 Garching, Bavaria, Germany

A. S. Bandarenka

Physics of Energy Conversion and Storage, Department of Physics, Technische Universität München (TUM), James-Franck-Str. 1, 85748 Garching, Germany.

Catalysis Research Center, TUM, Ernst-Otto-Fischer-Straße 1, 85748 Garching, Germany.

E-mail: bandarenka@ph.tum.de

Funding: Deutsche Forschungsgemeinschaft (DFG, German Research Foundation) under Germany's Excellence Strategy – EXC 2089/1 – 390776260 (e-conversion)

Keywords: Aqueous batteries, electrolytes, fast charging, potassium

**1. Structural Characterization of the Copper Hexacyanoferrate (CuHCF) Cathode**


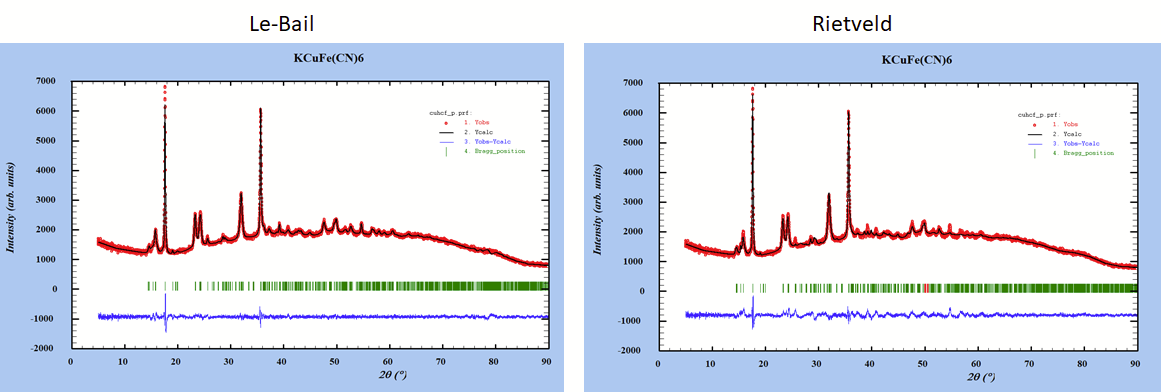


***Figure S1.*** *Fitted powder x-ray diffraction pattern of the CuHCF particles. The pattern corresponds to a monoclinic crystal structure with the lattice parameters shown in* ***Table S1.***

| **Parameter** | **Value** | **Error** |
| --- | --- | --- |
| a | 11.22637 Å | 0.00046 Å |
| b | 7.30188 Å | 0.00028 Å |
| c | 6.95463 Å | 0.00029 Å |
| α | 90.00000° | 0.00000° |
| β | 92.93085° | 0.00455° |
| γ | 90.00000° | 0.00000° |

***Table S1.*** *Cell parameters extracted from the fitted XRD spectrum of the CuHCF cathode material.*

**2. Structural Characterization of the Poly(Naphthalene Four Formyl Ethylenediamine) (PNFE) anode**

| (A)  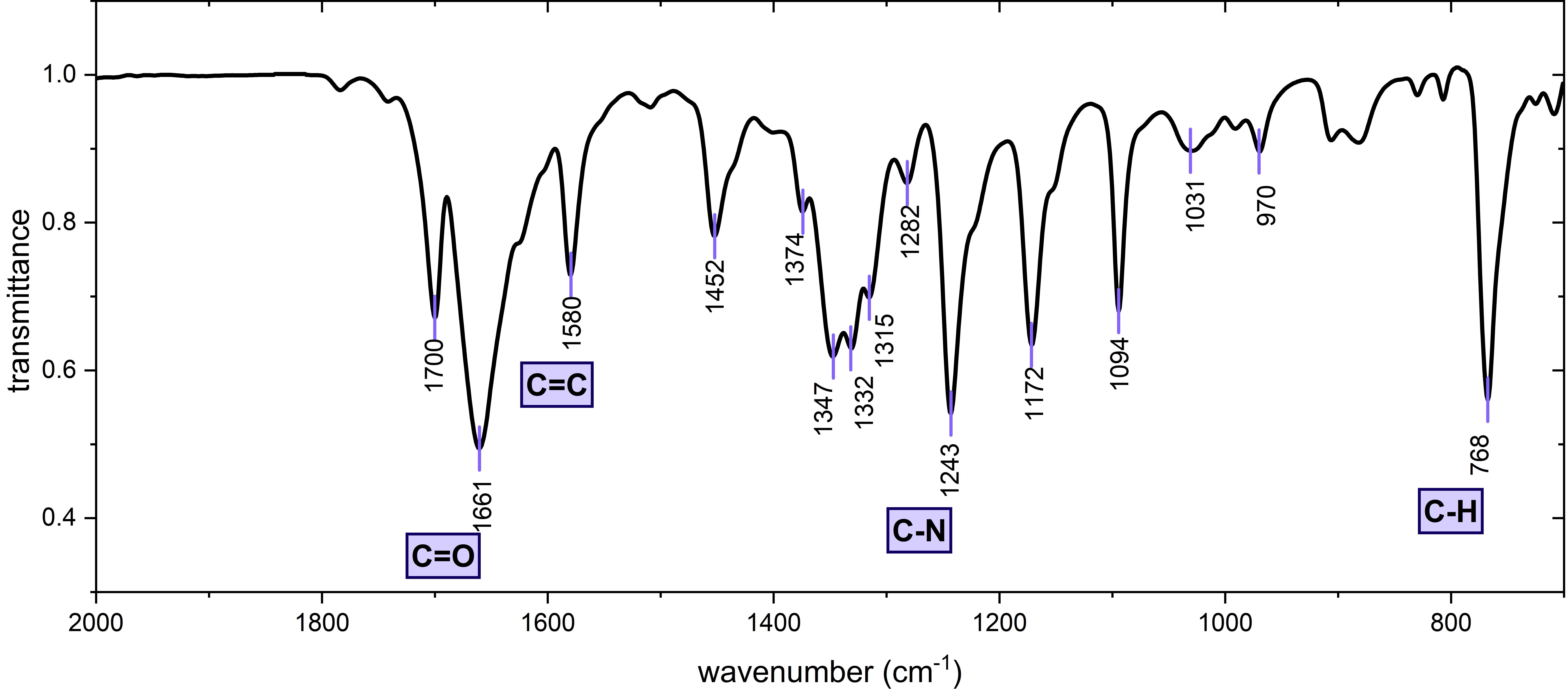 | |
| --- | --- |
| (B)  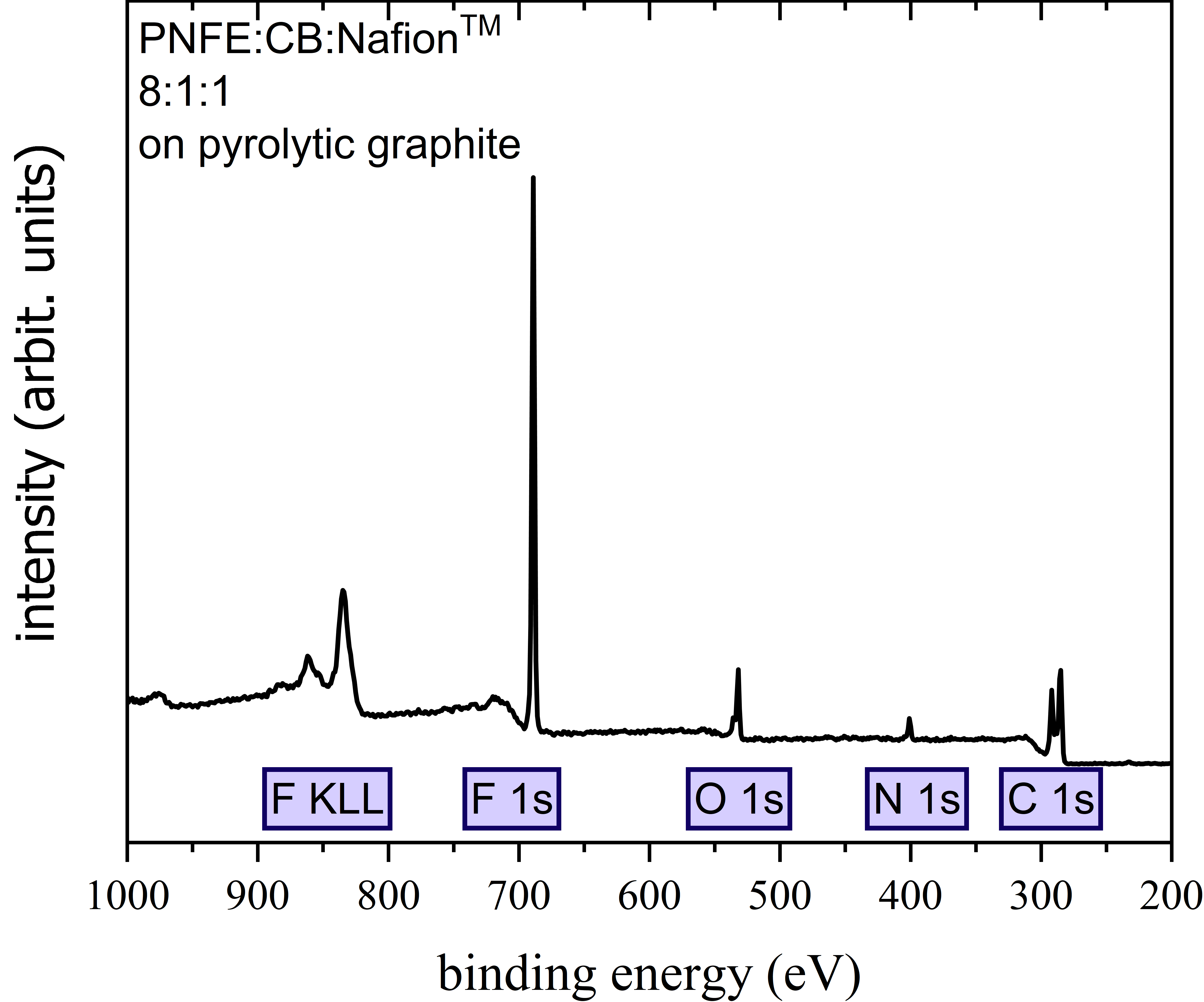 | (C)  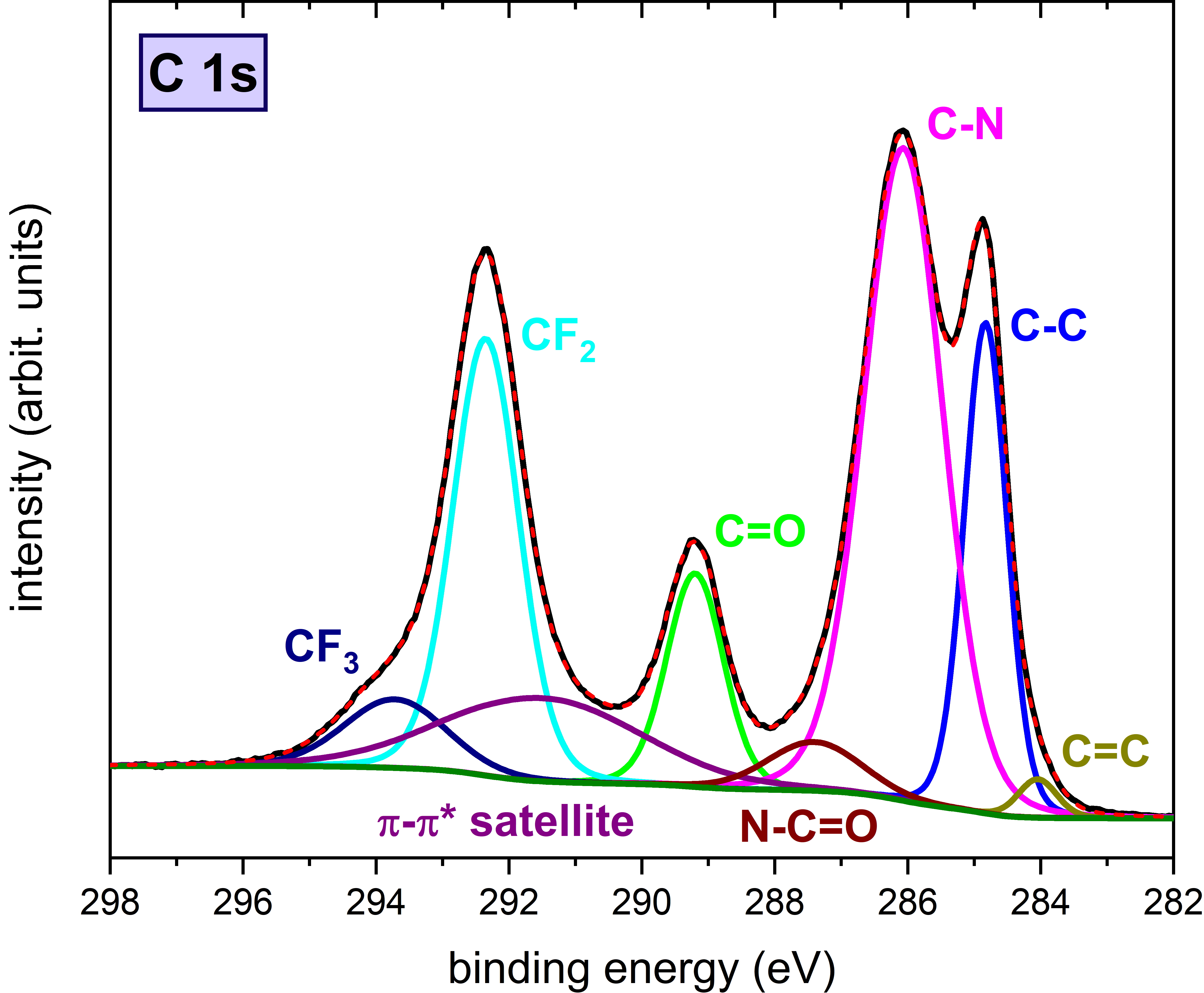 |

***Figure S2.*** *(A) FTIR spectrum of the PNFE electrode active material. (B) XPS survey scan of the PNFE anode. (C) Fitted C 1s XPS spectrum of the PNFE anode. (B) and (C) are replotted from our previous work. ^[1]^*

**3. BET Analysis of the Anode and Cathode**

| (A)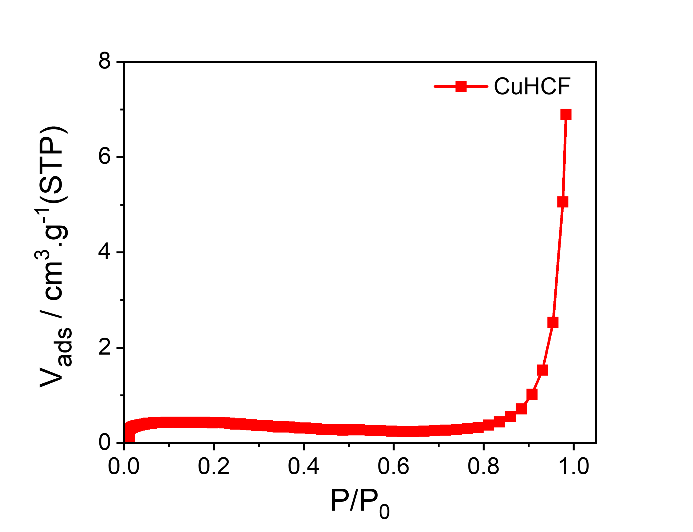 | (B)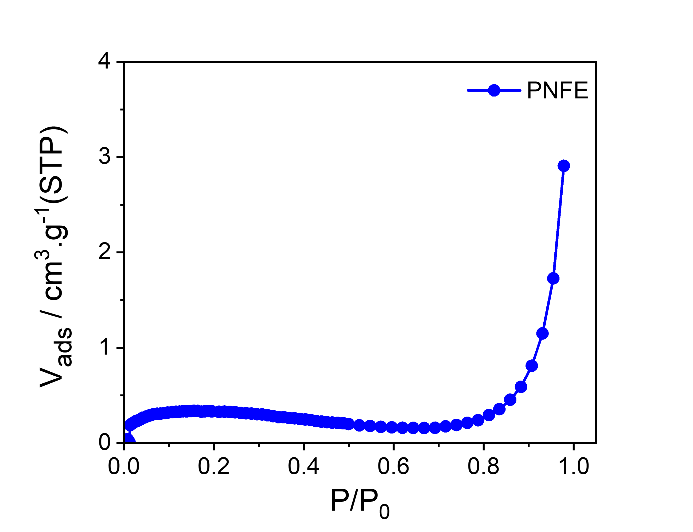 |
| --- | --- |

***Figure S3.*** *BET analysis of the anode and cathode obtained for N_2_ adsorption at 77 K. (A) Isotherm of the CuHCF cathode. (B) Isotherm of the PNFE anode.*

**4. Viscosity**

**
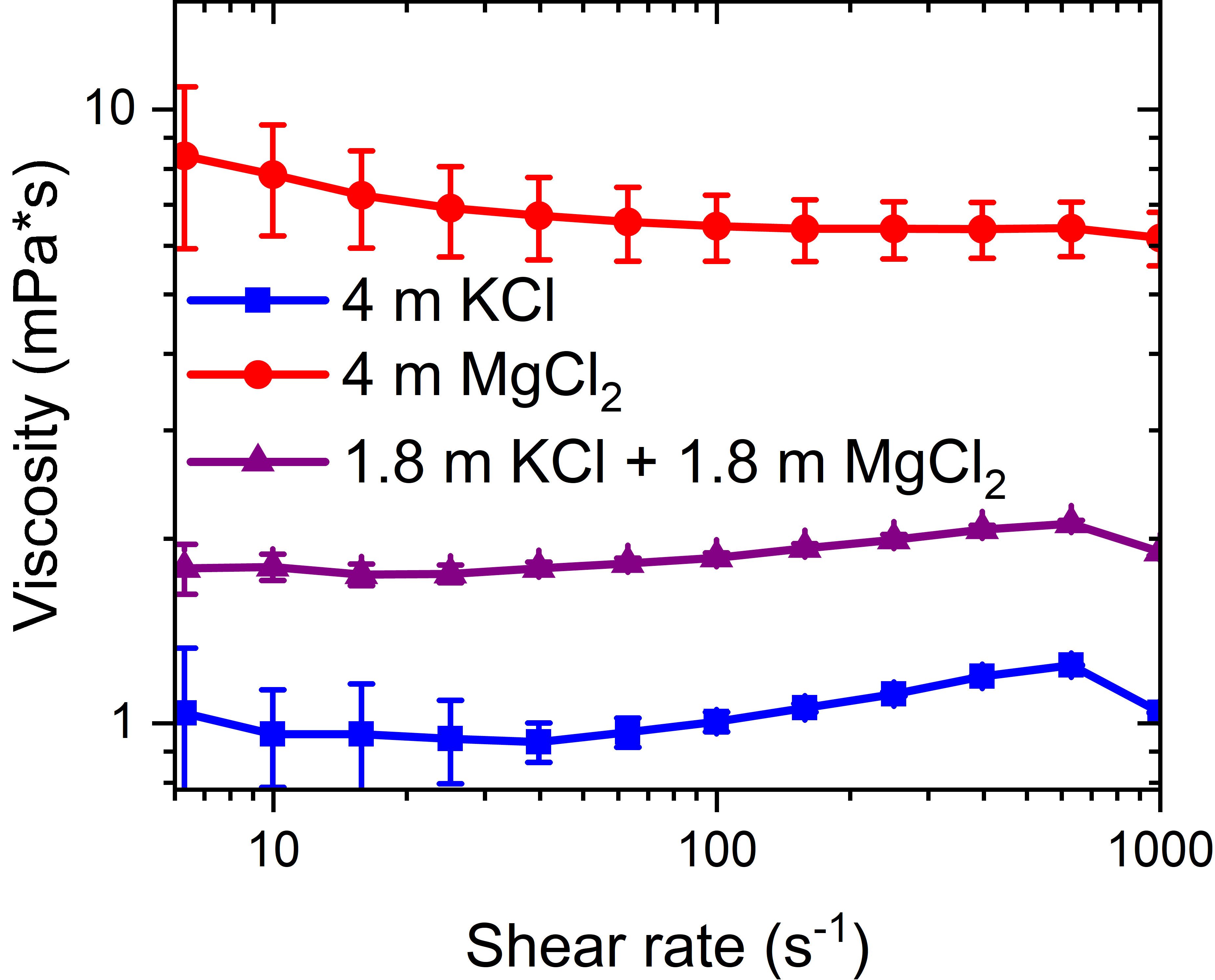
**

***Figure S4.*** *Viscosities of 4 m KCl, 4 m MgCl_2_, and 1.8 m KCl + 1.8 m MgCl_2_ measured at different shear rates at 25 °C.*

**5. Structure of the CuHCF Cathode Before and After Cycling**

| **(A)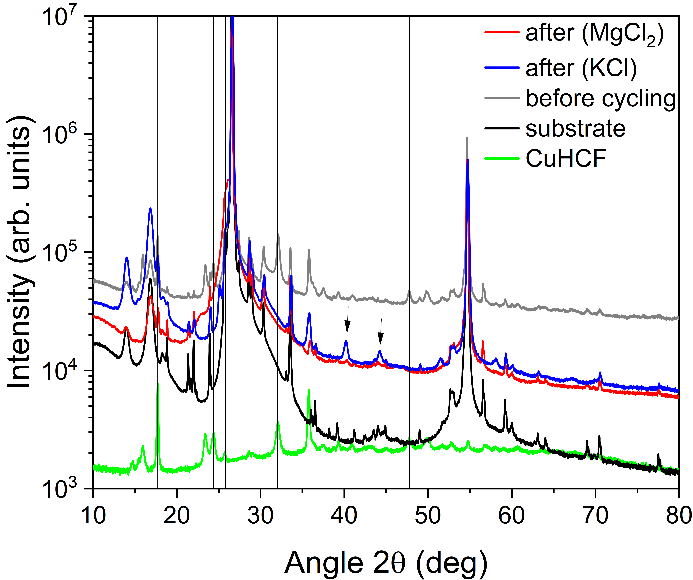** | | **(B)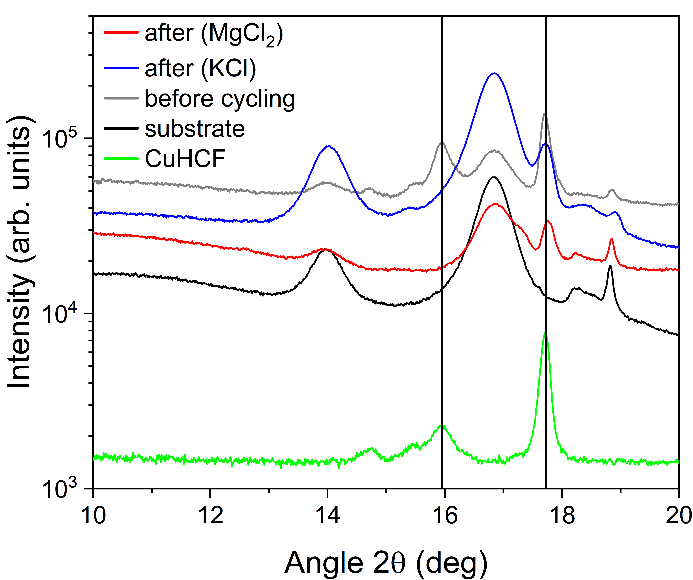** | |
| --- | --- | --- | --- |
| **(C)**  **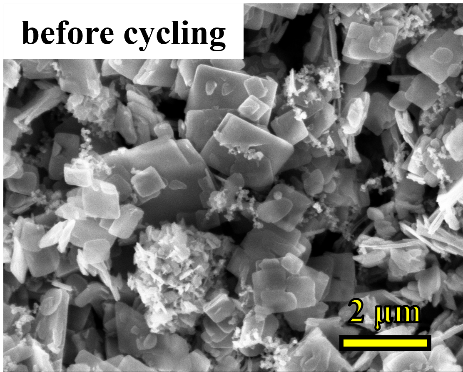** | **(D)**  **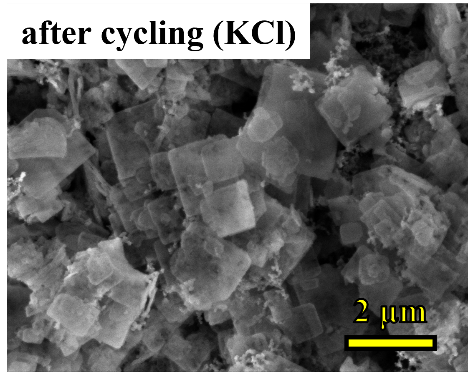** | | **(E)**  **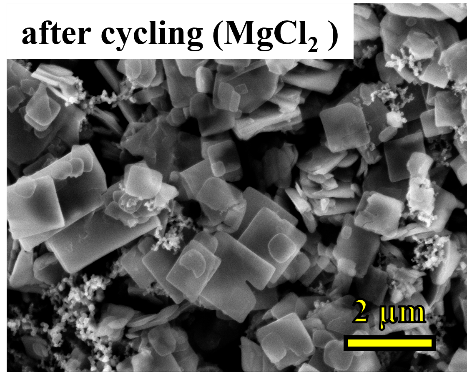** |

***Figure S5.*** *Structure of the CuHCF cathode before and after cycling. (A) XRD pattern of pure CuHCF (green), the CuHCF cathode (including the graphite-based substrate, carbon black and the PVDF binder, gray), the graphite-based substrate (black), the cathode after cycling in KCl (blue) and in MgCl_2_ (red). Arrows mark two superstructural peaks in the highly degraded KCl sample. (B) Zoom-in on the monoclinic (011) reflection, corresponding to the pseudo-cubic (200) reflection. (C) SEM image taken before cycling. (D) SEM image taken after cycling in KCl. (E) SEM image taken after cycling in MgCl_2_.*

**6. SEM-EDX Analysis of the CuHCF cathode reduced in the mixed KCl/MgCl_2_ electrolyte**

| (A)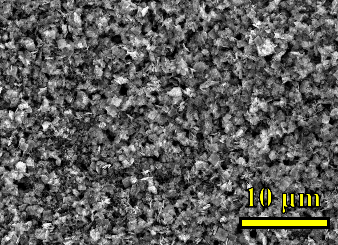 | (B)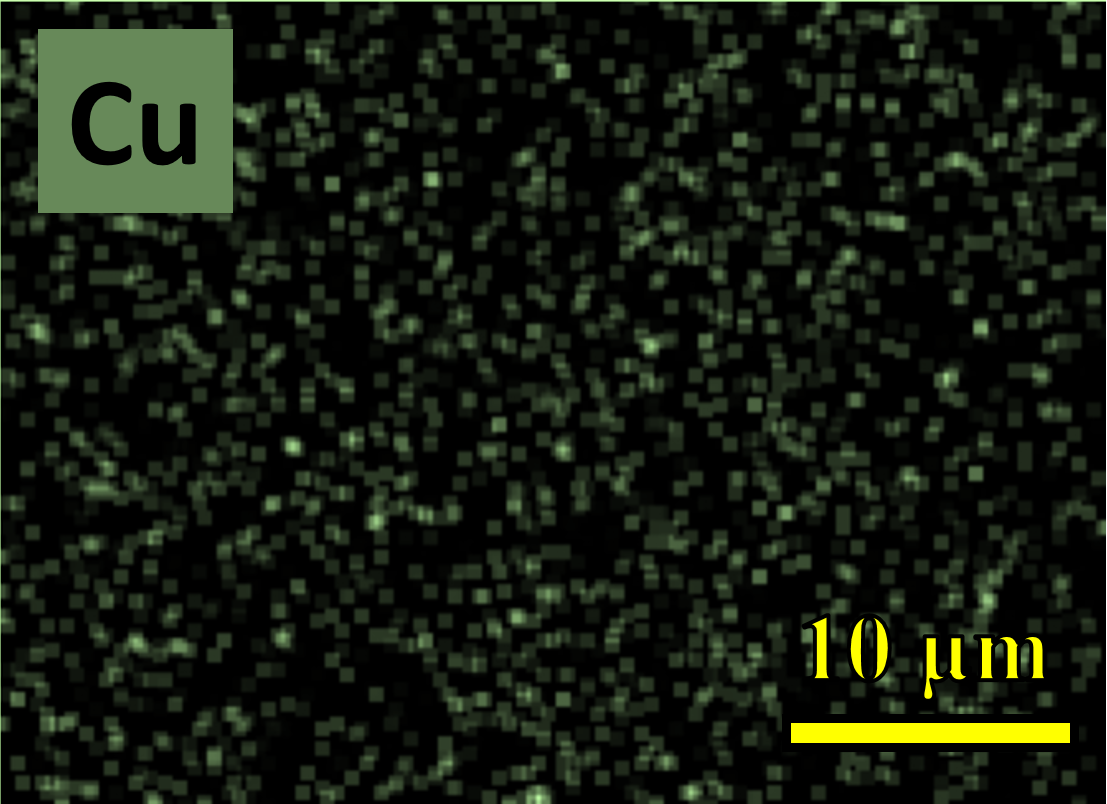 | (C)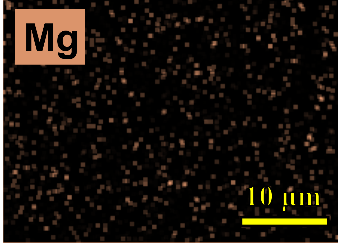 | (D)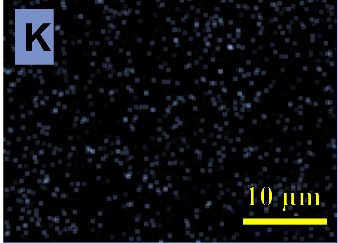 |
| --- | --- | --- | --- |

***Figure S6.*** *SEM-EDX elemental maps of the CuHCF cathode after being fully reduced in the mixed 1.8 m KCl + 1.8 m MgCl_2_ electrolyte. (A) SEM image of the investigated area. (B) elemental map for Cu-L. (C) Elemental map for Mg-K. (D) Elemental map for K-K.*

**7. SEM Analysis of the PNFE Anode Before and After Cycling in 4 m KCl**

| (A)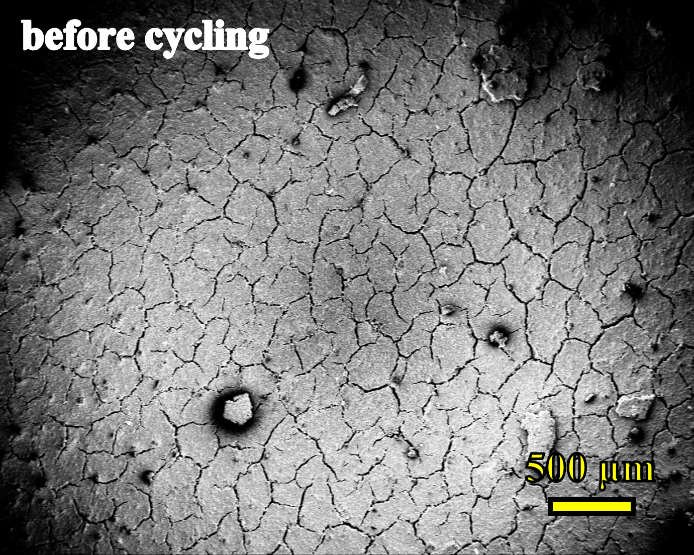 | (B)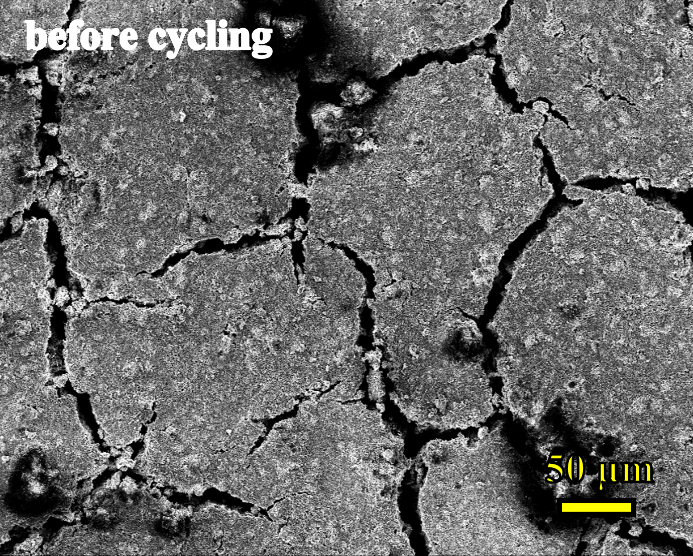 |
| --- | --- |
| (C)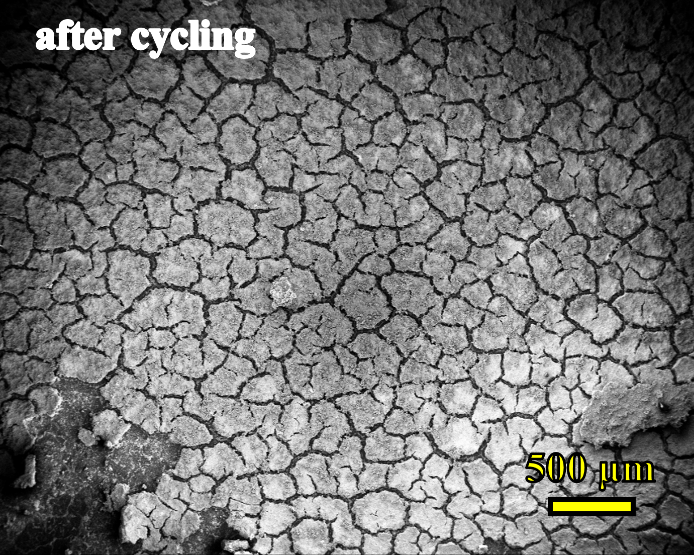 | (D)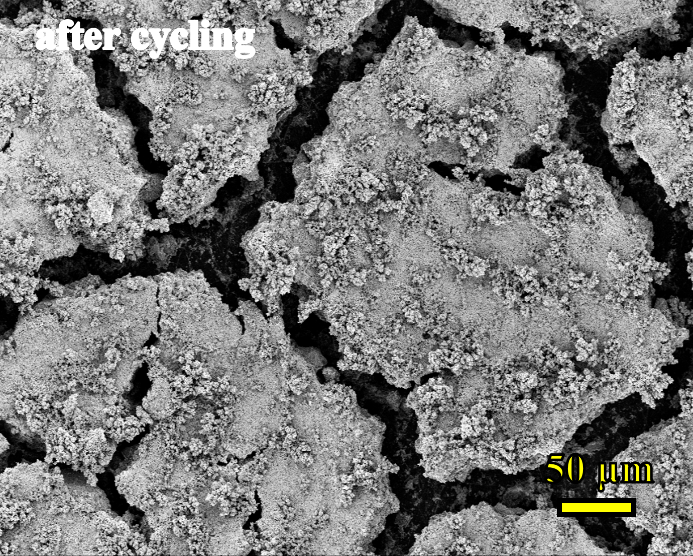 |

***Figure S7.*** *SEM analysis of the degradation of the PNFE anode in 4 m KCl. (A) SEM image at low magnification before cycling. (B) SEM image at higher magnification before cycling. (C) and (D) SEM images of the PNFE anode after cycling for 1000 cycles at 100 C in 4 m KCl.*

**References**

[1] Streng, R.L., Vagin, S., Guo, Y., Rieger, B., Bandarenka, A.S., Identifying the Charge Storage Mechanism in Polyimide Anodes for Na-Ion Aqueous Batteries by Impedance Spectroscopy, 2024, Energy Adv., 3, 4, 874–882, 10.1039/D4YA00037D.
